# Exploring Cultural Adaptations: A Scoping Review on Adolescent Mental Health and Substance Use Prevention Programs

**DOI:** 10.1007/s11121-025-01779-x

**Published:** 2025-01-31

**Authors:** Claudia Corpus-Espinosa, Isotta Mac Fadden, María del Carmen Torrejón-Guirado, Marta Lima-Serrano

**Affiliations:** 1 Faculty of Nursing, Physiotherapy, and Podiatry, Department of Nursing, Universidad de Sevilla, Avenzoar Street, 6, 41009 Seville, Spain; 2 Instituto de Biomedicina de Sevilla, IBiS/Hospital Universitario Virgen del Rocío/CSIC, Universidad de Sevilla, Seville, Spain; 3 Social Sciences Faculty, Department of Sociology, Universidad de Salamanca, Francisco Tomás y Valiente Avenue, no., 37071 Salamanca, Spain

**Keywords:** Cultural adaptation, Adolescence, Evidence-based interventions, Mental health promotion, Substance use

## Abstract

Adolescence is a critical period for developing risk behaviors such as substance use, which can impact health in adulthood. Culturally adapted evidence-based programs (EBPs) are promising for prevention. This review explores the processes for culturally adapting EBPs targeting alcohol, tobacco, or cannabis use, and/or anxiety or depression in adolescents. We searched multiple databases, grey literature, and relevant websites for studies detailing the cultural adaptation process of EBPs. We then categorized common adaptation steps, classified adaptations using the cultural sensitivity model, identified prevalent adaptation techniques, and determined effectiveness assessment methods. We reviewed the cultural adaptation processes of 43 EBPs. These programs were implemented in schools (51%), followed by family settings (30%), community settings (14%), and multi-component settings (5%). Eleven key steps were identified across the documents: local needs assessment, program selection, understanding of the program's curriculum, advisory group establishment, first draft of initial adaptation changes, staff selection and training, pilot study, enhanced cultural adaptation, implementation, evaluation and monitoring, and dissemination. Most programs integrated both surface (e.g., use of local images, material translation) and deep adaptations (e.g., incorporation of cultural values like "familismo"). Despite the common use of the cultural sensitivity model, detailed adaptation frameworks were often lacking. The field has advanced, but clearer documentation is needed to improve research and practical application.

**Supplementary Information:**

The online version contains supplementary material available at 10.1007/s11121-025-01779-x.

## Introduction

Throughout life, etiological factors—risk and protective—can emerge at various stages; however, those arising during critical developmental periods, such as childhood and adolescence, are particularly impactful (Reiner et al., 2019). During these key stages, the emergence of risk behaviors, such as substance use, can severely affect the quality of life, contributing to the development of chronic diseases and posing significant risks to mental health (Valenzuela-Mujica et al., 2013). These behaviors are often influenced by various risk factors, including peer pressure, unstable family environments, and lack of opportunities. However, protective factors, such as high self-esteem and parental supervision, can play a crucial role in counteracting these risks and promoting healthier developmental outcomes (Nawi et al., 2021).

In adolescence, substance use—particularly involving alcohol, tobacco, and cannabis—becomes a prevalent risk behavior with significant health consequences (Nebhinani et al., 2022). In 2021, the leading global disease burden, measured in Disability-Adjusted Life Years (DALYs), was attributed to behavioral risks, with alcohol use and smoking being the top contributors among adolescents (Brauer et al., 2024).

Research highlights a bidirectional relationship between mental health issues and substance use, where mental health problems can heighten the risk of substance use, and substance use can exacerbate mental health issues, with shared etiological factors and social determinants of health driving both (National Institutes of Health, 2024).

The World Health Organization (WHO) (2022) defines mental health as “mental health is a state of mental well-being that allows people to cope with life's stressful moments, develop all their abilities, learn and work adequately, and contribute to the improvement of their community.” Mental health is shaped by individual, social, and structural factors that can either protect or negatively influence it. Globally, approximately 14% of individuals aged 10 to 19 are affected by depression and anxiety (WHO, 2021), conditions commonly classified as "common mental health disorders," which rank among the leading causes of illness and disability (WHO, 2017).

To address this, implementing evidence-based interventions (EBIs) is crucial, especially in vulnerable contexts (Marsiglia et al., 2019). EBIs —defined as programs, practices, processes, policies, and guidelines with proven efficacy or effectiveness in a specific population and context (Rabin et al., 2008)—offer a cost-effective means to engage youth in preventive education (Marsiglia et al., 2022). More specifically, an evidence-based program (EBP) is a set of coordinated activities that demonstrate effectiveness on some desired outcomes and have been rigorously evaluated (Mihalic & Elliot, 2015).

Developing a new program from scratch requires significant resources, therefore, culturally adapted programs can enhance engagement and optimize resources by building upon proven programs (Marsiglia et al., 2019). Culturally adapting programs involve systematically modifying a program, including deletions or additions, changes in manner or intensity, and modifications to cultural aspects to fit and address the cultural beliefs, values, and practices of a specific population to ensure cultural relevance adoption, and acceptability of programs, which in turn increases participant engagement and attendance (Barrera, 2013). Several models have been proposed to guide the cultural adaptation of EBPs. These models can be divided into two categories: those focusing on the content of adaptations and those guiding the adaptation process.

Frameworks describing the content include Bernal’s (1995) Ecological Validity Model (EVM), which identifies eight dimensions to better meet the needs of the target population. The Cultural Sensitivity model by Resnicow et al. (1999) also emphasizes content, distinguishing between surface structure (enhancing acceptability through cultural elements) and deep structure (considering cultural, social, historical, and environmental, factors affecting health behavior). Another framework is the Psychotherapy Adaptation and Modification Framework (PAMF; Hwang, 2006), which outlines six domains for EBPs adaptation.

Frameworks describing the process of cultural adaptation include the following: Kumpfer et al. (2008) developed a model based on their experiences with the Strengthening Families Program, which consists of nine steps. Barrera and Castro (2006) introduced the Heuristic framework for the cultural adaptation of interventions, outlining several steps. Additionally, the ADAPT-ITT model also offers guidance (Montero-Zamora et al., 2021a), among others.

Overall, current initiatives have made significant strides in cultural adaptation; however, many existing frameworks exhibit inconsistencies and ambiguities, suggesting they may not fully encompass all the components of the adaptation process (Chu & Leino, 2017). A clearer understanding of these aspects would enhance study comparisons and enable a more comprehensive examination of the impact of adaptations. Furthermore, the literature highlights the importance of applying culturally adapted EBPs to prevent adolescent substance use and promote mental health (Chu & Leino, 2017; Lee et al., 2024).

A scoping review is a systematic type of knowledge to synthesize an existing or emerging body of literature on a specific topic, making it ideal for exploring the cultural adaptation of EBPs due to its broad coverage of diverse literature, which makes it optimal for topics with a heterogeneous research landscape and allows for the identification of knowledge gaps. This approach facilitates a comprehensive understanding of the adaptation processes while providing a foundation for future research in the field (Alves-Apóstolo, 2017). The research questions were: 1) What frameworks are employed in effecting cultural adaptations and what procedural steps do they have in common? 2) What types of adaptations are made? 3) What techniques are used to adapt the programs? and 4) How do authors evaluate whether the adaptation was successful or not? This scoping review marks one of the first attempts to explore cultural adaptations on a global scale for EBPs aimed at preventing substance use and common mental health problems among culturally diverse communities.

## Methods

### Study Design

The research questions were addressed using the scoping review methodology, following guidelines from the Joanna Briggs Institute (JBI) and PRISMA-ScR (Peters et al., 2015; Tricco et al., 2018). The protocol was registered with the Open Science Framework on 20/06/2023 (https://osf.io/5n37e/).

### Eligibility Criteria

The PCC (Participants, Concept, Context) eligibility criteria are as follows (Peters et al., 2015):

- Population: The study focused on adolescents (10–19 years old) (WHO, 2023a). To broaden the scope, the search strategy also included keywords "children" and "preadolescents," using thesaurus headings and relevant scientific article keywords.
- Concept: The study concentrated on methodologies or models for culturally adapting EBPs targeting the prevention of common mental health disorders (specifically: anxiety and depression) and substance use (specifically: alcohol, tobacco, or cannabis) whether individually or collectively.
- Context: The context was considered as the setting where the program was implemented, i.e., at school, family, or community level.

We included documents employing qualitative, quantitative, and mixed methodologies if they provided details on the adaptation process or if such information was retrievable. Studies were included regardless of publication period, language, or source type.

Exclusion criteria included papers lacking explicitly stating that the program was evidence-based. To verify the original and adapted programs' based on evidence, we applied the JBI levels of evidence for effectiveness. These levels establish a hierarchy for assessing the quality of evidence regarding the effectiveness of programs, with higher levels indicating stronger evidence (JBI, 2013). Additionally, papers lacking details on the cultural adaptation process or methodology were excluded.

### Information Sources

Literature research was conducted from February 2023 to October 2024 across various bibliographic databases, including PubMed, Scopus, PsycINFO, Embase, Web of Science, CINAHL, Cochrane, OpenGrey, and websites such as WHO, Pan American Health Organization (PAHO), Substance Abuse and Mental Health Services Administration (SAMHSA), National Institute on Alcohol Abuse and Alcoholism (NIAAA), National Institute on Drug Abuse (NIDA) and the European Monitoring Centre for Drugs and Drug Addiction (EMCDDA). Search strategies were developed using MeSH and DeCS terms, CINAHL subject headings, and free-text terms. Strategies were refined with librarian guidance and team discussions. Reference lists of included articles, systematic reviews, and scoping reviews were also examined for additional papers. No filters were applied during database searches. Final search strategies for Scopus, as an example, can be found in Online resource 1.

### Study Selection and Data Extraction

The search results were exported to Zotero (Cohen et al., 2023) for reference management, and duplicate entries were removed. Titles, abstracts, and keywords of identified documents were examined, and those not meeting inclusion criteria were excluded. One researcher (CACE) conducted initial screening, with subsequent reviews and verifications by two reviewers (IMF and MLS). Consensus was sought to resolve disagreements. Documents passing initial screening were transferred to the collaborative platform "rayyan" (Ouzzani et al., n.d.) for two independent reviewers to examine them (MCTG and CACE); the consensus was sought to resolve disagreements (MLS). Those not meeting inclusion criteria were excluded.

A data-charting form was developed collaboratively in Excel by the researchers to extract variables. This form included details such as the name of the EBP, adapted program, home and adaptation countries, program objectives, target population, facilitators, context, cultural adaptation framework, theoretical foundation, key adaptation steps, types of adaptations, techniques used, and adaptation effectiveness. One researcher charted the data (CACE), with two reviewers (IMF and MLS) validating the results through discussions and seeking consensus in case of disagreements. The form underwent continuous updating iteratively.

To address the interrogations posed above, we examined the frameworks or methodologies documented in the studies for culturally adapting the programs, the steps entailed by these methodologies or models, the nature of adaptations implemented, whether superficial or deep, the techniques employed for adapting the EBP´s, and the evaluations conducted to assess the effectiveness of the adapted program.

### Synthesis of Results

The articles were linked based on several factors: authorship, where the content of each document was examined for connections; retrospective references, where, if an evaluation article of an adapted program was identified, citation and reference searches were conducted to link related articles comprehensively; and prospective references, where, if an adaptation article was found, the authors were checked for any mention of ongoing evaluation studies or related work. Regardless of whether such studies were mentioned, The program name was searched in the previously mentioned databases without applying filters. This linking process was initially conducted by one researcher (CACE) and subsequently reviewed by two additional researchers (MFI and MLS). In cases of disagreement, consensus was reached.

In this review, which included multiple study designs, a segregated design was employed following the methodology described by Marshall et al. (2021). This approach allowed qualitative and quantitative data to be considered separately, yet in a complementary manner, to address the research questions. Qualitative data were used to explore the cultural adaptation processes and the social validity of the programs, where applicable, as part of the reported outcomes from each adapted program in the included documents. Meanwhile, in response to the fourth research question of this scoping review, and considering the diverse nature of the documents, quantitative data were examined to summarize the outcomes, based on the authors’ reported results. These health-related outcomes varied by program and included measures such as attitudes, knowledge, skills, and behaviors, all reported as part of the program results. Data from this review were synthesized and presented narratively to condense and elucidate the key findings.

A content analysis approach was used, guided by two frameworks for adapting EBPs (Barrera & Castro, 2006; Kumpfer et al., 2008), to identify common program adaptation steps. Descriptions for each step were developed based on the authors' accounts. The frequency of each step's inclusion and the use of frameworks were determined. A final table was compiled and cross-verified by two reviewers (MLS and IMF).

Program adaptation types were categorized as superficial or deep using Resnicow et al.'s model. Surface adaptations involve adjusting materials and messages to the target population's observable characteristics (e.g., language, food, music, locations), while deep adaptations focus on understanding the cultural, social, historical, environmental, and psychological factors influencing the target health behavior. Additionally, two literature reviews that classify cultural adaptations based on this model were utilized to guide the classification process (Marshall et al., 2021). Based on this, adaptations were classified as superficial when they involved modifications of elements such as language, places, music, food, games, images, or symbols that make the program more familiar or culturally recognizable. They were classified as deep when they addressed cultural constructs, such as values, beliefs, or social norms that influence behavior. This includes adaptations that consider gender roles, family structures, or community practices that are deeply embedded in the culture of the target population.

One researcher (CACE) classified each adaptation, and subsequently, two additional researchers evaluated this classification (MFI and MLS). In cases of disagreement, consensus was sought.

The most utilized techniques (focus groups, interviews, etc.) for program adaptation or evaluation were identified. Lastly, the assessment of program effectiveness was determined.

## Results

### Study Selection

**Fig. 1:**
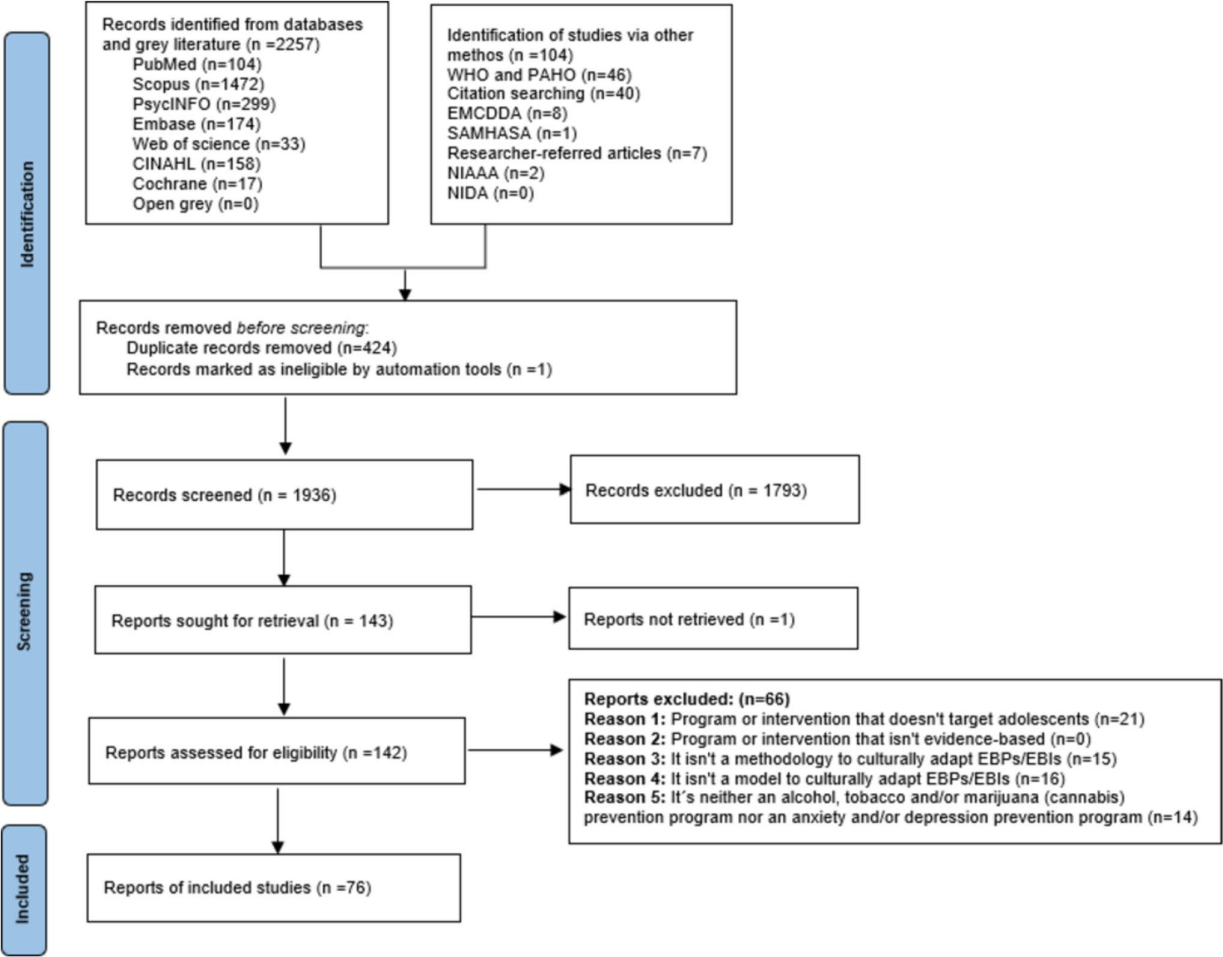
PRISMA flow diagram

A total of 2,361 studies were compiled, and after removing duplicates and excluding citations marked as ineligible by automated tools, 1,936 remained. Following the screening of titles and abstracts, 143 full-text articles were assessed, with 66 excluded based on the criteria outlined in Fig. 1. One study was excluded due to unavailability. Ultimately, 76 studies were included in the review (Fig. 1). A full list of the studies included for review can be found in Online Resource 2.

### Program Characteristics

The included articles describe 43 adapted programs, the majority of which originated in the USA (33), followed by the UK (6), Australia (1), Puerto Rico (1), Europe (1), and the Netherlands (1). Most programs were implemented in school settings (n = 22), followed by family settings (n = 13), community settings (n = 6), and two multi-component settings involving school, family, and community.

**Fig. 2:**
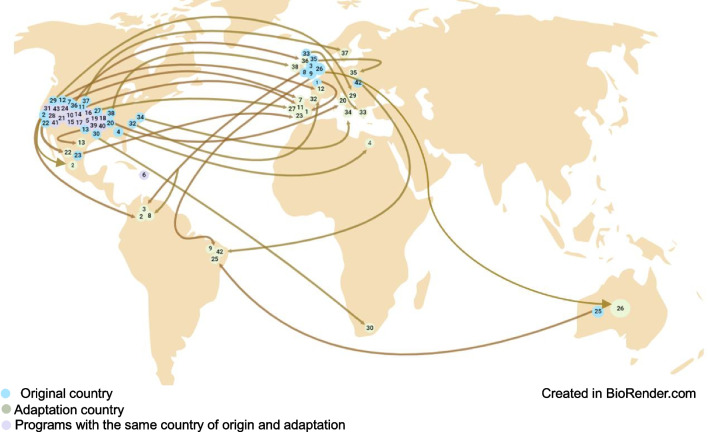
Cultural adaptation processes distribution

As seen in Fig. 2, most of the adaptation processes were conducted within the USA, with shifts in target populations, such as from urban to rural areas or from American to Latino populations. Similar processes were observed more frequently in European countries. However, there were fewer reported instances of culturally adapted programs in lower-middle-income countries.

Additional characteristics of these programs are provided in Online resource 2. The attributes were chosen based on findings reported in consulted studies (Escoffery et al., 2018; Movsisyan et al., 2021).

### Cultural Adaptation Frameworks In Prevention Programs

The process of culturally adapting the programs has been variable; as shown in Table 1 while some programs use a single framework (CATCH-IT, EMAS, Familias Fuertes, Familias Fuertes Brazil, GGC, ITC, Jóvenes Fuertes, KiR Pennsylvania and Ohio, KiR 5th grade version, L2W, NTC, RAD-PAL, SFP 10–14 Italy and Poland, SFP 12–16, Slick Tracy Home, TALKnTIME), others use two to complement the cultural adaptation (Bacanísimo, CURB, Entre Parceros, MREAL Mexico, RISE, TG, UTC), and in some cases, even three (PN). Nevertheless, among the 43 EBPs, 18 studies did not mention whether they employed a specific model or not (Alerta Alcohol, ASPIRE, Familias que funcionan Spain, Familien stärken, Entrenamiento en Habilidades de Vida, KiR LGBT + community, KiR Texas, LST, MREAL Spain, PERAE, Preventure, Project EX, PN Croatia, SFP 10–14 Greece, Sweden and UK, SFP France, #Tamojunto).

**Table 1:** Cultural adaptations of included evidence-based programs

N°	Program	Reported steps		Reported framework	Effectiveness evaluation
1	Alerta Alcohol (Lima-Serrano et al., 2018;Martínez-Montilla et al., 2022 ; Martínez-Montilla, 2020; Vargas-Martínez et al., 2023) *Netherlands (Dutch) → Spain (Spanish)*	1.Local needs assessment 2.Program selection 3. Understanding the curriculum 4.Advisory group	7. Pilot 8.Enhanced cultural adaptation 9. Implementation 10. Evaluation	N/A	Cluster RCT
2	ASPIRE (Tamí-Maury et al., 2019) *USA (Spanish) → Colombia/Mexico (standard Spanish)*	4.Advisory group 5.Initial adaptation 7.Pilot	8.Enhanced cultural adaptation 10. Evaluation	N/A	Pre-posttest design
3	Bacanísimo (Sánchez-Franco et al., 2021) *UK (English) → Colombia (Spanish)*	1.Local needs assessment 4.Advisory group 5.Initial adaptation 6. Training	7. Pilot 8.Enhanced cultural adaptation 10. Evaluation	Heuristic framework FRAME	Pre-posttest design
4	CATCH-IT (Abuwalla et al., 2019) *USA (English) → Arab Countries (An Arabic dialect)*	1.Local needs assessment 4.Advisory group (experts)	5.Initial adaptation	PEN-3 cultural model	N/A
5	CURB (Bansa et al., 2018; Saulsberry et al., 2013) *USA (English) → Chicago, USA (adolescents in English parents in Spanish)*	1.Local needs assessment 4.Advisory group (target population)	5.Initial adaptation	PEN-3 cultural model Strategies for enhancing cultural appropriateness	RCT with wait-list
6	EMAS (Sáez-Santiago et al., 2017) *Puerto Rico (Spanish) → Puerto Rico (Spanish)*	4.Advisory group 5.Initial adaptation 6. Training	7. Pilot 8.Enhanced cultural adaptation 10. Evaluation	Ecological validity model	Pilot study
7	Entrenamiento en Habilidades de Vida (De los Ángeles Luengo Martín et al., 1999) *USA (English) → Spain (Spanish)*	2.Program selection 3. Understanding the curriculum 4.Advisory group 5.Initial adaptation	6. Training 7. Pilot 8.Enhanced cultural adaptation 9.Implementation 10. Evaluation	N/A^c^	Quasi-experimental design
8	Entre Parceros (Sánchez-Franco et al., 2021) *UK (English) → Colombia (Spanish)*	1.Local needs assessment 4.Advisory group 5.Initial adaptation 6. Training	7.Pilot 8.Enhanced cultural adaptation 10. Evaluation	Heuristic framework FRAME	Pre-posttest design
9	Familias Fuertes Brazil(Abreu et al., 2021; Murta et al., 2018, 2020, 2021; Sanchez et al., 2024) *UK (English) → Brazil (Portuguese)*	1.Local needs assessment 2.Program selection 3. Understanding the curriculum 4.Advisory group 5.Initial adaptation	6. Training 7. Pilot 8.Enhanced cultural adaptation 9.Implementation 10. Evaluation 11. Dissemination	SFP's cultural adaptation recommendations	Cluster RCT
10	Familias Fuertes Latin American immigrant (Azziz-Baumgartner C & Wilson, 2009; Orpinas et al., 2014) *USA (English) → USA (adolescents in English parents in Spanish)*	3. Understanding the curriculum 4.Advisory group 5.Initial adaptation	6. Training 9. Implementation 10. Evaluation	Guidelines for Balancing Program Fidelity/Adaptation	N/A
11	Familias que funcionan Spain (Pérez et al., 2010) *USA (English) → Spain (Spanish)*	4.Advisory group (experts) 5.Initial adaptation	7.Pilot 8.Enhanced cultural adaptation 10. Evaluation	N/A	Longitudinal analysis at 1 and 2 years
12	Familien stärken (Baldus et al., 2016; Bröning et al., 2014; Stolle et al., 2011) USA (English) → Germany (English)	4.Advisory group 6. Training	7.Pilot 10. Evaluation	N/A	Multi-center RCT
13	GGC (Montero-Zamora et al., 2021a, 2021b, 2022) *USA (English) → Mexico (Spanish)*	1.Local needs assessment 2.Program selection 4.Advisory group 5.Initial adaptation	6. Training 7.Pilot 8.Enhanced cultural adaptation 10. Evaluation	ADAPT-IT	Quasi-experimental pilot design
14	ITC (Baldwin et al., 2021, Lowe, 2024) *USA (Tribal language) → USA (Tribal language)*	1.Local needs assessment 4.Advisory group 6. Training	8.Enhanced cultural adaptation 9.Implementation 10.Evaluation	Circular Model of Cultural Tailoring	Quasi-experimental study
15	Jóvenes Fuertes (Castro-Olivo & Merrell, 2012) *USA (English) → USA, Latino Immigrant (Spanish)*	4.Advisory group 7.Pilot	8.Enhanced cultural adaptation 10. Evaluation	Ecological validity model	Pre-posttest design
16	KiR LGBT + community (Goldbach & Holleran Steiker, 2011) *USA (English) → USA (English)*	1.Local needs assessment 3.Understanding the curriculum 4.Advisory group (target population)	5.Initial adaptation 6. Training 7.Pilot 8.Enhanced cultural adaptation	N/A	N/A
17	KiR Pennsylvania and Ohio (Colby et al., 2013; Hecht et al., 2018) *USA (English) → USA (English)*	1.Local needs assessment 3. Understanding the curriculum 4.Advisory group 5.Initial adaptation	6. Training 7.Pilot 8.Enhanced cultural adaptation 9. Implementation 10. Evaluation	Cultural sensitivity model	RCT
18	KiR Texas (Holleran Steiker et al., 2014) *USA (English) → USA (English)*	1.Local needs assessment 3. Understanding the curriculum 4.Advisory group (target population)	5.Initial adaptation 6. Training 7.Pilot 8.Enhanced cultural adaptation 10. Evaluation	N/A	Quasi-experimental design
19	KiR 5th grade version (Harthun et al., 2009; Hecht et al., 2008) *USA (English) → USA (English)*	1.Local needs assessment 3. Understanding the curriculum 4.Advisory group 5.Initial adaptation	6. Training 7.Pilot 8.Enhanced cultural adaptation 10. Evaluation	CBPR	Longitudinal RCT
20	LST (Velasco et al., 2015, 2017) *USA (English) → Italy (Italian)*	2.Program selection 4.Advisory group 5.Initial adaptation 6. Training 7.Pilot	8.Enhanced cultural adaptation 9.Implementation 10. Evaluation 11. Dissemination	N/A	Quasi-experimental design
21	L2W (Jumper-Reeves et al., 2013; Kulis et al., 2016) *USA (English) → USA (N/A)*	1.Local needs assessment 3. Understanding the curriculum 4.Advisory group 5.Initial adaptation	6. Training 7.Pilot 8.Enhanced cultural adaptation 9. Implementation 10. Evaluation	CBPR	RCT
22	MREAL Mexico (Kulis et al., 2021; Marsiglia et al., 2019, 2022) *USA (English) → Mexico (Spanish)*	1.Local needs assessment 2.Program selection 3. Understanding the curriculum 4.Advisory group 5.Initial adaptation	6. Training 7.Pilot 8.Enhanced cultural adaptation 9.Implementation 10. Evaluation	Ecological validity model Cultural sensitivity model	Cluster RCT
23	MREAL Spain (Cutrín et al., 2021; Cutrín et al., 2022) *Mexico (Spanish) → Spain (Spanish)*	1.Local needs assessment 2.Program selection 3. Understanding the curriculum 4.Advisory group 5.Initial adaptation	6. Training 7.Pilot 8.Enhanced cultural adaptation 9.Implementation 10. Evaluation	N/A	RCT
24	NTC (Patchell, 2011) *USA (Tribal language) → USA (Tribal language)*	1.Local needs assessment 2.Program selection 3. Understanding the curriculum 4.Advisory group	6. Training 8.Enhanced cultural adaptation 10. Evaluation	Circular Model of Cultural Tailoring	Pre-posttest design
25	PERAE (Amato et al., 2021; De Castro-Amato, 2015) *Australia (English) → Brazil (Portuguese)*	4.Advisory group 5.Initial adaptation 6. Training	7.Pilot 8.Enhanced cultural adaptation 10. Evaluation	N/A	Pilot RCT
26	Preventure (Barrett et al., 2015; Debenham et al., 2021) *UK (English) → Australia (English)*	1.Local needs assessment 4.Advisory group 5.Initial adaptation	8.Enhanced cultural adaptation 10. Evaluation	N/A	Cluster RCT
27	Project Ex (Espada et al., 2014) *USA (English) → Spain (Spanish)*	3. Understanding the curriculum 4.Advisory group 5.Initial adaptation	6. Training 10. Evaluation	N/A	RCT
28	PN (Komro, 2004; Komro et al., 2008) *USA (English) → USA (English)*	1.Local needs assessment 2.Program selection 3. Understanding the curriculum 4.Advisory group	6. Training 7.Pilot 8.Enhanced cultural adaptation 9.Implementation 10. Evaluation	Cultural sensitivity model, The theory of triadic influence, Perry´s planning model for adolescent health promotion programs	RCT
29	PN Croatia (Abatemarco et al., 2004; West et al., 2008) *USA (English) → Croatia (Croatian)*	1.Local needs assessment 2.Program selection 3. Understanding the curriculum 4.Advisory group 5. Initial adaptation	6. Training 8.Enhanced cultural adaptation 9.Implementation 10. Evaluation	N/A	RCT
30	RAD-PAL (Carney et al., 2020a, 2020b) *USA (English) → South Africa (N/A)*	1.Local needs assessment 2.Program selection 4.Advisory group 5.Initial adaptation	6. Training 8.Enhanced cultural adaptation 10. Evaluation	ADAPT-IT	Single-arm trial
31	RISE (Clarke et al., 2022) *USA (English) → USA, African Americans (English)*	4.Advisory group 5.Initial adaptation 6. Training	7.Pilot 8.Enhanced cultural adaptation 10. Evaluation	CBPR Cultural sensitivity model	Pilot community trial
32	SFP France (Roehrig & Pradier, 2017) *USA (English)*)* → France (French)*	3. Understanding the curriculum 4.Advisory group 5.Initial adaptation 6. Training	7.Pilot 8.Enhanced cultural adaptation 9.Implementation 10. Evaluation 11.Dissemination	N/A	Pilot study
33	SFP 10–14 Greece (Kyritsi & Bacopoulou, 2021) *UK (English) → Greece (Greek)*	4.Advisory group 5.Initial adaptation 6. Training	7.Pilot 8.Enhanced cultural adaptation 10. Evaluation	N/A	Exploratory trial
34	SFP 10–14 Italy (Ortega et al., 2012) *USA (English) → Italy(Italian)*	1.Local needs assessment 2.Program selection 4.Advisory group 5.Initial adaptation 6. Training	8.Enhanced cultural adaptation 9.Implemetation 10. Evaluation 11.Dissemintation	SFP's cultural adaptation recommendations	Pilot study
35	SFP 10–14 Poland (Foxcroft et al., 2016; Okulicz-Kozaryn & Dorozko, 2008) *UK (English) → Poland(Polish)*	1.Local needs assessment 2.Program selection 4.Advisory group 5.Initial adaptation 6. Training	7.Pilot 8.Enhanced cultural adaptation 9.Implemetation 10. Evaluation	SFP's cultural adaptation recommendations	Cluster RCT
36	SFP 10–14 UK (Allen et al., 2007; Segrott et al., 2022) *USA (English) → UK (English)*	4.Advisory group 5.Initial adaptation 6. Training	7.Pilot 10. Evaluation	N/A	Pragmatic cluster-RCT
37	SFP 10–14 Sweden (Skärstrand et al., 2008, 2014) *USA (English) → Sweden (Swedish)*	4.Advisory group 5.Initial adaptation 6. Training	7.Pilot 10. Evaluation	N/A	RCT
38	SFP 12–16 (Kumpfer et al., 2012) *USA (English) → Ireland (English)*	1.Local needs assessment 2.Program selection 4.Advisory group 5.Initial adaptation 6. Training	7.Pilot 8.Enhanced cultural adaptation 10. Evaluation 11.Dissemination	SFP's cultural adaptation recommendations	Quasi-experimental design
39	Slick Tracy Home (Komro et al., 2006) *USA (English) → USA (Spanish, Chinese, Polish)*	4.Advisory group 5.Initial adaptation 6. Training 7.Pilot	8.Enhanced cultural adaptation 9.Implementation 10. Evaluation	Cultural sensitivity model	RCT
40	TALKnTIME (Noël, 2014; Noël et al., 2013) *USA (English) → USA (English)*	3. Understanding the curriculum 4.Advisory group 5.Initial adaptation	6. Training 10.Evaluation	PAR	RCT
41	TG (Asdigian et al., 2023; Ivanich et al., 2020; Whitesell et al., 2019) *USA (English) → USA (English and a tribal language)*	2.Program selection 4.Advisory group 5.Initial adaptation	6. Training 7.Pilot 10. Evaluation	MOST approach CBPR	Pre-posttest design
42	#Tamojunto (Medeiros et al., 2016; Pedroso & Hamann, 2019; Sanchez et al., 2021) *Europe (N/A) → Brazil (Portuguese)*	4.Advisory group 5.Initial adaptation 6. Training	7.Pilot 9.Implementation 10.Evaluation	N/A	RCT
43	UTC (Wimbish-Cirilo, 2016) *USA (Tribal language) → USA (Tribal language)*	1.Local needs assessment 4.Advisory group 6. Training	8.Enhanced cultural adaptation 9.Implementation 10.Evaluation	CBPR Look, Think, Act action research cycle	Quasi-experimental design

PAR: Participatory Action Qualitative Research; RCT: Randomized controlled trial; N/A: Not available, indicating that it was not mentioned in the document; CBPR: Community−based participatory research; FRAME: Framework to Report Adaptation and Modifications−Expanded; MOST: Multiphase optimization strategy

Note: the names of the steps shown here are not presented in full. The complete names can be found in the main text, Fig. 2, and Online Supplementary Material 4

The most commonly used methodology was the cultural sensitivity model reported 5 times (KiR Pennsylvania and Ohio, MREAL Mexico, PN, RISE, and Slick Tracy Home), followed by the ecological validity model (EMAS, Jóvenes Fuertes, MREAL Mexico) and the third most employed were the Community Based Participatory Research (CBPR) (KiR 5th grade version, L2W, RISE, UTC), and the SFP´s recommendations for cultural adaptations (Familias Fuertes Brazil, SFP 10–14 Italy and Poland, and SFP 12–16).

Frameworks that were also reported for adapting the EBPs include: Participatory Action Qualitative Research (PAQR) for the TALKnTIME program, The Look, Think, Act action research cycle for UTC, the Circular Model of Cultural Tailoring for ITC and NTC, the PEN-3 cultural model for CATCH-IT and CURB, in the latter, also used the Strategies for Enhancing Cultural Appropriateness, the MOST approach and a Community Engaged Process to adapt TG, the Heuristic framework for the cultural adaptation of interventions and FRAME for Entre Parceros and Bacanísimo, the ADAPT-ITT framework for RAD-PAL and GGC, the Guidelines for Balancing Program Fidelity/Adaptation for Familias Fuertes and The theory of triadic influence and Perry´s planning model for adolescent health promotion programs for PN.

The review highlighted significant variability in how adaptation processes were documented. While all studies provided information on their adaptation process, the level of detail varied considerably. Documents focused on program adaptation, such as Familias Fuertes Brazil, MREAL in Mexico, GGC, SFP 10–14 Poland, L2W, and PN, provided more comprehensive details of the processes followed. In contrast, documents focused on evaluation, like MREAL Spain, KiR Texas, and Project X, presented the adaptation steps in a more summarized manner. This discrepancy suggests that reported steps may not fully reflect actual practices. Greater transparency in future research is needed to ensure clear and thorough documentation of methodologies.

### Cultural Adaptation Stages and Process Description

**Fig. 3:**
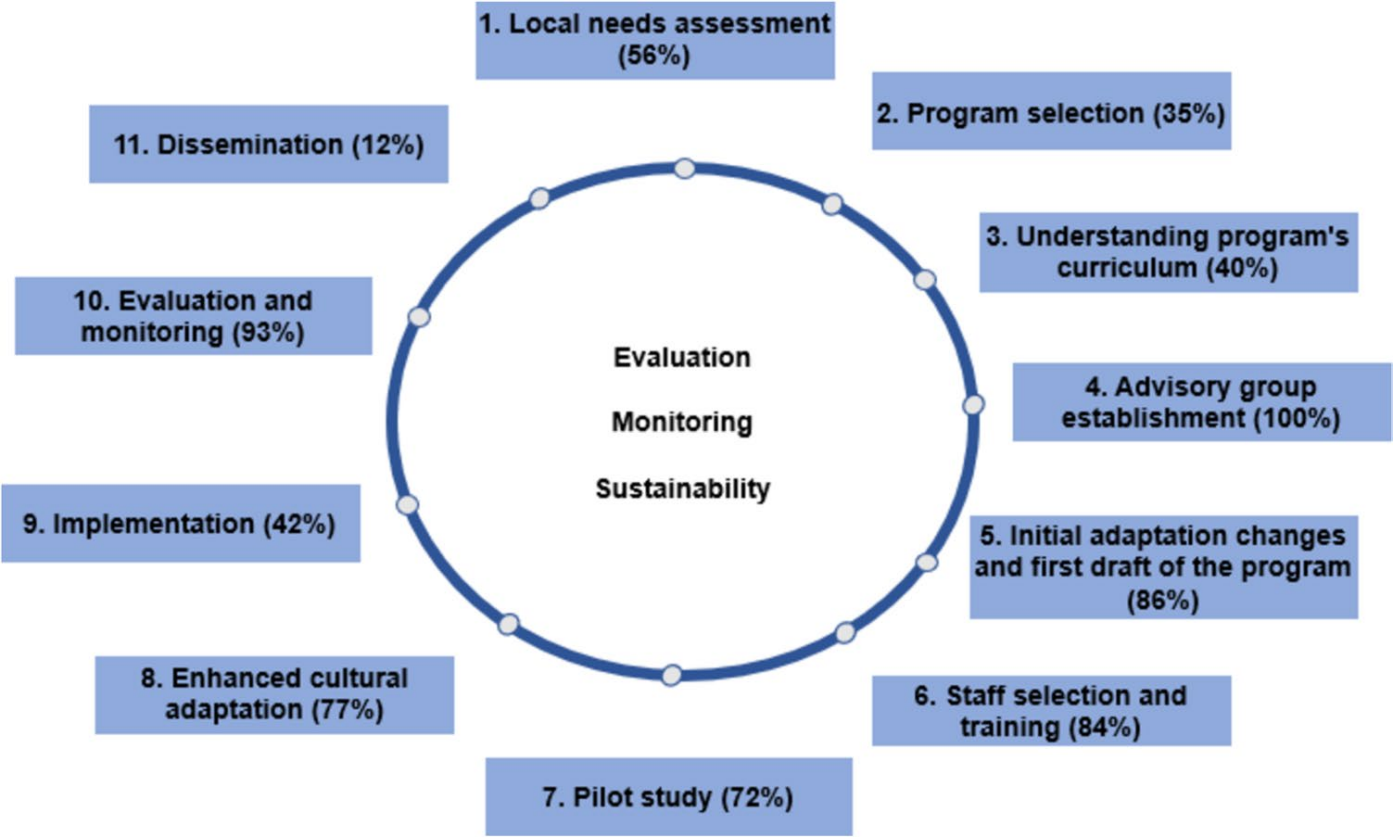
Iterative nature of cultural adaptation process

The cultural adaptation processes comprised distinct yet occasionally overlapping steps. We delineated 11 steps in the adaptation of programs, which we classified into the following categories (Fig. 3).

The analysis revealed that a crucial step emphasized in multiple studies is understanding participants' characteristics, preferences, and values, commonly known as “local needs assessment” (Table 1). This assessment influences outcomes such as receptivity, adherence, and overall effectiveness, and it also addresses risk and protective factors as well as psychosocial challenges related to substance use and mental health. As shown in Fig. 2, only 56% of the reviewed studies reported conducting this assessment before adaptation. Some programs implemented regular reviews and updates to maintain the relevance and effectiveness of their programs, underscoring the importance of continuous refinement to meet emerging needs and enhance overall impact. This step was executed by: Alerta Alcohol, Bacanísimo, CATCH-IT, CURB, Entre Parceros, Familias Fuertes Brazil, GGC, ITC, KiR LGBT + community, L2W, MREAL Mexico and Spain KiR Pennsylvania and Ohio, KiR Texas, KiR 5th grade version, NTC, Preventure, PN, PN Croatia, RAD-PAL, SFP 10–14 Italy and Poland, SFP 12–16 and UTC.

Another step documented by only a few programs is “program selection” (35%), which involves collecting information on existing EBPs and selecting those that align with the community’s needs and context. For example, Familias Fuertes Brazil, MREAL Mexico and Spain, NTC, PN, PN Croatia, SFP 10–14 Italy and Poland, SFP 12–16, were among those that followed this step (Table 1).

An additional aspect that received limited attention in the studies included in this review was “understanding the program's curriculum,” with only 40% addressing it. This is concerning since comprehending the theoretical foundations of the program and its core components is crucial to avoid potential impacts on the program's effectiveness. This action was performed by: Alerta Alcohol, Entrenamiento en Habilidades de Vida, Familias Fuertes, Familias Fuertes Brazil, KiR LGBT + community, KiR Pennsylvania and Ohio, KiR Texas, KiR 5th grade version, L2W, MREAL Mexico and Spain, among others (Table 1).

The most prevalent step in all adaptation processes (100%) was the “advisory group establishment”, typically composed of a mix of experts and members from the target community and stakeholders. Experts often encompassed original curriculum developers, mental health experts, public health experts, drug field specialists, cultural adaptation experts, researchers, among others. On the other hand, the target population and stakeholder group commonly consisted of parents, adolescents, community leaders, teachers, families, and other relevant members. In some cases, both expert and target population advisory groups were established, while in others, only the target population was involved in the cultural adaptation process (CURB, KiR LGBT + community, KiR Texas). To a lesser extent, some adaptations exclusively involved the experts group (CATCH-IT, Familias que funcionan) Throughout the cultural adaptation process, this advisory group provided continuous support, integrating contributions from evaluations, the advisory group provided continuous support.

The subsequent most frequently reported step (86%) involved the creation of an initial draft. During this phase, consideration was given to the potential adaptation or non-adaptation of the program and to making preliminary adjustments to materials and sessions, such as translation, back-translation, subtitling, or dubbing, as needed to better align with the contextual nuances. Emphasis was placed on verifying whether any alterations would impact the core components.

The adaptation processes where these aspects are evident are: Alerta Alcohol, ASPIRE, Bacanísimo, CATCH-IT, CURB, EMAS, Entre Parceros, Entrenamiento en Habilidades de Vida, Familias Fuertes, Familias Fuertes Brazil, GGC, KiR LGBT + community, KiR Pennsylvania and Ohio, KiR Texas, KiR 5th grade version, LST, MREAL Mexico and Spain, including other programs, as shown in Table 1.

The next step, and also one of the most frequently implemented, was “staff selection and training” (84%), referring to the process of identifying and preparing the individuals responsible for program implementation. These individuals typically undergo training facilitated by a group of certified professionals or by the authors of the EBPs to ensure implementation quality. Moreover, the training is also adapted to the context. The following outlines some cultural adaptation processes related to this step, with further details available in Table 1: EMAS, Entrenamiento en Habilidades de Vida, Familias Fuertes Brazil, Familien stärken, GCC, ITC, KiR Texas, LST, MREAL Mexico and Spain, NTC, PERAE, Project EX, PN, RAD-PAL, RISE, SFP France, SFP 10–14 Greece, Italy, Poland Sweden and UK, etc.

The next step, reported by 72% of the adapted EBPs, was the pilot phase, in which the program was implemented with a small group of participants. The goals were to review and modify elements as needed while preserving core components to maintain effectiveness, and to collect data on retention rates, enrollment, fidelity, and social validation to identify and address barriers in the process.

The pilot phase enabled the identification of improvements for future cultural adaptations based on evaluations from the target population and stakeholder feedback. This allowed for adjustments such as modifying session durations, adapting language to participants' educational levels, and incorporating additional sessions or content. The subsequent list presents several cultural adaptation processes relevant to this step, with additional details provided in Table 1: Alerta Alcohol, ASPIRE, Bacanísimo, EMAS, Entre Parceros, Familias Fuertes Brazil, Familias que funcionan, Familien stärken, GGC, Jóvenes Fuertes, KiR Pennsylvania and Ohio, KiR Texas, etc.

Regarding step 8, this was documented in 77% of the programs, wherein, building upon preceding steps, new adaptations were implemented aimed at enhancing the program's cultural sensitivity (Table 1). It is important to note that these programs emphasized cultural adaptation as an iterative process. For instance, in programs such as Familias Fuertes Brazil or Entrenamiento en habilidades de Vida, ongoing evaluations were conducted to ensure the program remained culturally relevant to the target population. This step, along with others, could be repeated to ensure the program was closely aligned with the participants' culture and representative of their context, without modifying its core components. Through multiple feedback cycles, the programs were refined before advancing to the refined before advancing to the next phase, "implementation" (42%).

In the "evaluation and monitoring" step (95%), the second most reported step, ongoing data gathering, and analysis were conducted to track progress toward the porgram's objectives. This included direct observation of selected sessions by the research team, external observers, and trained personnel, who provided feedback to facilitators. Facilitators also completed forms, checklists, and field diaries after each session. These instruments and procedures, used individually or in combination, aimed to assess the fidelity and quality of program implementation. On the other hand, evaluation involved generating evidence of the program's effects to establish associations between the EBP and expected health outcomes. In addition to assessing effectiveness, it was crucial to evaluate the social validation of the program concerning its feasibility, acceptability, satisfaction and perceived utility for both facilitators and the target audience. Despite their distinct nature, these evaluations are interrelated and offer a comprehensive and holistic view of program performance. Grouping them emphasizes their combined role in understanding the program’s impact and implementation (EMCDDA, 2011).

As previously mentioned, ongoing evaluations consistently gathered feedback from participants and stakeholders to assess the need for program modifications, propose suggestions for future adaptations, or determine suitability for scaling to other populations. Several programs implementing this stage include those listed, with all programs detailed in Table 1: Alerta Alcohol, ASPIRE, Entre Parceros, EMAS, Familias Fuertes Brazil, Familias que funcionan, Familien stärken, GCC, ITC, Jóvenes Fuertes, KiR 5th grade version, KiR Pennsylvania and Ohio, KiR Texas, LST, MREAL Mexico and Spain, NTC, PERAE.

Finally, the least reported step was “dissemination” (12%), involving training system development for broader dissemination efforts, alongside ongoing program readaptation and reevaluation. This was performed by: Familias Fuertes Brazil, LST, SFP France, SFP 10–14 Italy, and SFP 12–16.

It is noteworthy that while Online resource 3 may present the cultural adaptation process as linear and orderly, this is not always the case; it is actually iterative, with steps often occurring concurrently and factors like evaluation, sustainability, and involving the target population and stakeholders should be continually considered to guide the adaptation process (Fig. 3).

### Kinds of Cultural Adaptations

While some documents offered comprehensive descriptions, others provided less detail, potentially influenced by the adaptation stage, study objectives, journal limitations, and the type of document. Nonetheless, all documents detailed the modifications made. Among the 43 culturally adapted EBPs, 27 (63%) incorporated surface and deep structural adaptations, while 16 (38%) focused solely on surface adaptations. Common surface adaptations included language refinement, translation, visual adjustments, and modifications to make activities and examples more familiar to adolescents. In contrast, deep adaptations typically involve integrating contextual values, history, and beliefs, that influence health (Online Resource 4 provides examples of adaptations made in the programs).

### Adaptation Techniques

Most adaptation processes in EBPs involve two to five techniques, comprising both qualitative and quantitative methods. However, seven programs, including Familias Fuertes, Familias que funcionan, Familien stärken, GCC, KiR LGBT + community, L2W, and Project EX, utilized only qualitative techniques such as focus groups, nominal groups, interviews, etc. Collaboration between community members and experts was common, with some programs adapted solely from the experts' perspective (Abuwalla et al., 2019; Pérez et al., 2010) and others exclusively from the viewpoint of the target population and stakeholders (Goldbach & Holleran Steiker, 2011; Holleran Steiker et al., 2014; Saulsberry et al., 2013). While project leadership was not consistently explicit, available information suggests that investigators likely led both the projects and the cultural adaptations of the included EBPs. Online resource 4 offers examples of adaptation techniques.

### Effectiveness Assessment of the Adaptations

Of the 43 EBPs analyzed, 40 conducted studies to evaluate the effectiveness of cultural adaptations. However, 3 EBPs either did not provide information on evaluation or did not make it the focus of the selected documents for this investigation (KiR LGBT + community, Familias Fuertes, and CATCH-IT).

Randomized controlled trials (RCTs) were used in 20 programs, with 11 demonstrating effectiveness for culturally adapted EBPs: Alerta Alcohol, KiR Pennsylvania and Ohio, L2W, MREAL Mexico and Spain, Preventure, PN Croatia, Project EX, RAD-PAL, Slick Tracy Home, TALKnTIME, as shown in Table 1, further details about this aspect can be found in Online resource 4.

In the UK and Germany, adapted programs showed no significant differences from control groups in substance use, emotional well-being, stress, or behavioral problems. Additionally, CURB's potential benefits were unproven due to insufficient trial participants, and the short-term effects of the KiR 5th-grade version were inconsistent. Similarly, three adaptation processes were found to be ineffective, particularly the SFP 10–14 program in Sweden, which underwent a complete structural change from a family-focused implementation to a school setting with minimal parental involvement. This shift occurred because the original program format did not align with the context. Such instances highlight the necessity of thoroughly understanding the curriculum, preserving the core components, and evaluating the feasibility of program implementation in a specific context before proceeding, as outlined in Steps 2 and 3 of the adaptation process. Moreover, the SFP 10–14 Poland adaptation had no impact on substance use outcomes, parenting skills, parent–child relationships, or child problem behaviors. Additionally, one adaptation, Tamojunto, resulted in an iatrogenic effect, underscoring the importance of preserving core components during the adaptation process.

In the case of Project Northland higher student attrition reduced exposure to the program. Over time, no significant differences in alcohol or drug use were observed between the intervention and control groups. The authors suggested that the social and environmental context may have influenced the program's effectiveness, as many community members did not view alcohol use as a priority concern. Furthermore, in urban areas with a high proportion of families living in poverty and increased exposure to alcohol outlets and advertisements, longer-term and more intensive efforts may be necessary, including making substantial changes to the environment.

Quasi-experimental studies were used in 18 programs, as detailed in Table 1. Out of these, 12 reported positive outcomes (ASPIRE, EMAS, Entrenamiento en Habilidades de Vida, GCC, ITC, Jóvenes Fuertes, KiR Texas, LST, NTC, SFP France, SFP 12–16, TG, UTC). Regarding the remaining five adapted programs, one study's results are unpublished (SFP Greece), while two encountered issues with family participation, preventing piloting in one (SFP Italy) and, despite obtaining favorable results in the other, authors acknowledge the need for replication with a larger sample (SFP Spain). The remaining two programs (Bacanísismo and Entre Parceros) showed potential, demonstrating feasibility and social adaptability in preliminary tests. However, the authors emphasize the need for further studies to evaluate their effectiveness.

Among the EBPs analyzed, six reported cost-effectiveness studies. Out of these, four were deemed cost-effective (Marsiglia et al., 2022; Vargas-Martínez, 2023; Whitesell et al., 2019; Wimbish-Cirilo, 2016), one was potentially cost-effective (Tamí-Maury et al., 2019), and one was not cost-effective(Segrott et al., 2022).

Based on the JBI levels of evidence for effectiveness, we evaluated both the original programs and their adapted versions. The vast majority of the original programs achieved a Level 1 or 2 evidence rating, indicating that their effectiveness was assessed through RCTs or quasi-experimental studies, respectively. Additionally, we found that 87% of the adapted programs also had a Level 1 or 2 evidence rating (Online resource 4).

## Discussion

The scoping comprehensively explored the types of adaptation, adaptation techniques, frameworks, and evaluations in 43 EBPs for preventing substance use and common mental health disorders, thus providing an overview of the adaptation process. This study will inform the subsequent stages of creating a methodological framework to guide the adaptation process.

Like in a prior scoping review, this study identified 11 steps for culturally adapting EBPs (Escoffery et al., 2018); moreover, this review demonstrates the iterative nature of the cultural adaptation process, delineates the diverse techniques applicable in adapting an EBPs, outlines recommended assessment methods, identifies stakeholders essential for this process, and provides examples of cultural adaptations implemented across the EBPs discussed herein. Additionally, it presents a compilation of various frameworks for cultural adaptation, contributing to literature. The frameworks used for program adaptation varied, with some processes not explicitly reporting any specific model. Documentation of the adaptation process also varied, with studies focusing on program adaptation offering more detailed reporting. Conversely, papers primarily focused on adaptation effectiveness tended to summarize the cultural adaptation process. This observation aligns with prior research emphasizing the importance of rigorous and consistent reporting based on scientific knowledge, which may be lacking due to the maturing nature of the field of cultural adaptation of EBPs (Chu & Leino, 2017; Movsisyan et al., 2021; Thier et al., 2019).

Across all the programs reviewed, establishing an advisory group during cultural adaptation was a consistent step (All programs reported completing this step). This aligns with existing research emphasizing the involvement of experts and stakeholders, underscoring the centrality of their participation (Martínez-Montilla, 2020; Movsisyan et al., 2021). Furthermore, this study identified a diverse range of individuals who can participate in this group. This serves as a reference for health professionals and researchers, indicating whom they can consult during cultural adaptation.

Moreover, many countries worldwide lack access to effective and culturally appropriate substance use prevention programs (Marsiglia et al., 2022). Low- and middle-income countries (LMICs) often have limited or no access to EBPs, while some prevention programs exist, most lack evidence of efficacy (Nadkarni et al., 2022). This gap can be attributed to inadequate healthcare access, insufficient training among facilitators, limited human resources, political and financial constraints, and competing priorities (Nadkarni et al., 2022). In contrast, high-income countries invest significantly more in prevention programs (WHO, 2023b). Moving forward, LMICs need to establish effective strategic planning in collaboration with authorities and policymakers, focusing on evidence-based decision-making, financial sustainability, long-term implementation, culturally adapted programs, and collaboration with stakeholders (Marsiglia et al., 2022).

### Strengths, Implications, and Limitations

This review exhibits several strengths, including the exploration of grey literature, and the inclusive approach of considering studies in any language. These methodological choices enhanced access to a substantial volume of research and facilitated the analysis of the adaptation process across diverse contexts and countries, addressing notable gaps in the existing literature. However, while the results of this review provide valuable insights into the "how" (steps), "what" (types of adaptations), and the techniques involved in culturally adapting prevention programs, there remain gaps in the level of detail reported for each stage of this process. This aspect is crucial for enhancing replicability, comprehending research nuances, assessing impact, and facilitating learning from these adaptations. Notably, just two programs were identified that referred to frameworks offering recommendations for reporting the cultural adaptation process (FRAME). Additionally, the underreporting of the use of adaptation frameworks in some studies suggests a potential for wider dissemination to inform future adaptation efforts.

An important aspect consistently highlighted across various programs is the identification of core components. Preserving these elements is crucial to prevent potential impacts on program effectiveness. Some studies mentioned involving original program authors to safeguard these components. However, not all authors' processes were identified. Therefore, conducting fidelity assessments becomes necessary and beneficial, along with understanding the program curriculum (Step 3). Hence, further research is warranted to maintain the core components and uphold program integrity. Additionally, reporting these aspects in articles would be valuable for future adaptation endeavors.

Regarding the assessment of effectiveness, the findings of this review align with prior research that underscores the necessity for conducting robust effectiveness trials of culturally adapted programs. These trials are essential to ascertain whether the observed effects are better or worse when compared to the original program. Furthermore, certain researchers have encouraged the utilization of RCTs as they are considered the most rigorous research design for evaluating the effectiveness of behavioral programs (Escoffery et al., 2018; Marshall et al., 2021).

This study encounters limitations stemming from variations in cultural adaptation approaches, diverse study designs, and distinct data presentation methods making it difficult to compare the results. Additionally, the researchers did not contact the authors of the included studies to gather information on the adaptations made, consequently, certain steps in the process may have been undertaken, yet the details were not reported. This may be due to factors such as space limitations in scientific publications, which may lead to the omission of certain key details. Furthermore, the type of document analyzed may also influence the depth of reporting, with these generally providing more exhaustive information compared to other types of publications. Consequently, it is important to emphasize the need to interpret the reported steps with caution, as the steps reported here do not necessarily reflect the steps used in practice, but rather as documentation of practices reported in the available literature. By documenting these gaps, this review contributes to a deeper understanding of the limitations of the available literature, emphasizing the importance of transparent and comprehensive information for future research on cultural adaptation.

On the other hand, the search strategies were vetted by a librarian, which enhances the credibility of the findings. However, the search strings employed may have constrained the search, potentially excluding articles that use alternative terminology.

The objective of this review was not to assess the effectiveness of the adapted programs themselves but to explore whether and how their effectiveness was evaluated as part of the adaptation process. Therefore, we summarized only the outcomes reported by the original authors. This approach was necessary because some adapted programs report their effectiveness based on health outcomes and social validation, which includes factors such as feasibility, acceptability, and usefulness, rather than directly assessing the efficacy of the adaptation methods employed. It is important to note that this lack of direct evaluation of the effectiveness of cultural adaptation methods represents a significant gap in the literature. This gap underscores the need for future studies to evaluate how these adaptation methods influence the success of EBPs. Finally, the quality assessment of the studies was omitted as per JBI guidelines for scoping reviews. Future systematic reviews might prioritize high-evidence studies to assess adapted programs´ efficacy.

Future research should explore the complexities of adapting and implementing EBPs for the prevention of substance use, depression, and anxiety, particularly in the context of marginalized adolescents. This focus is essential for addressing health inequities and enhancing engagement, program uptake, and health outcomes. Marginalized youth, especially those growing up in poverty, experience mental health issues at rates 2–3 times higher than the general population. (United Nations Office on Drugs and Crime, 2022).

This review serves multiple purposes. Firstly, it documents the most commonly reported steps in the cultural adaptation process found in the literature. Secondly, it presents various methodological models, program adaptation techniques, assessment methods, and EBPs aimed at preventing substance use, depression, and anxiety in adolescents across different contexts, including face-to-face and web-based programs. Furthermore, it identifies gaps in knowledge, highlighting areas for further research.

## Conclusion

This review has identified eleven common steps across various adaptation processes (local needs assessment, program selection, understanding program´s curriculum, advisory group establishment, first draft of initial adaptation changes., staff selection and training, pilot study, enhanced cultural adaptation, implementation, evaluation and monitoring and dissemination). However, there remains a need for more meticulous reporting, systematization, and detail of the adaptation process. Subsequent research efforts could benefit from the development of an adaptation model to systematically guide this process. Additionally, the findings underscore the importance of documenting the adaptation of programs for both research and practical applications. Finally, further investigation is needed into culturally adapted EBPs addressing substance use and common mental health disorders in adolescents from vulnerable communities. These insights will inform the next steps of developing a methodological framework to adapt EBPs focused on preventing common mental health disorders and substance use in adolescents living in vulnerable areas.

## Supplementary Information

Below is the link to the electronic supplementary material.

Supplementary file1 (PDF 121 KB)

Supplementary file2 (PDF 289 KB)

Supplementary file3 (PDF 189 KB)

Supplementary file4 (PDF 133 KB)

Supplementary file5 (PDF 129 KB)
