# Supplementary material for: Exploring Cultural Adaptations: A Scoping Review on Adolescent Mental Health and Substance Use Prevention Programs
Source: Prev Sci. 2025 Jan 31;26(2):204–21. doi: 10.1007/s11121-025-01779-x (PMC11891097; doi:10.1007/s11121-025-01779-x)
Supplement: Supplementary file 1 — Supplementary file1 (PDF 121 KB) [file 11121_2025_1779_MOESM1_ESM.pdf]

Authors: Claudia Corpus-Espinosa, Isotta Mac Fadden*, María del Carmen Torrejón-Guirado, Marta Lima-Serrano
*** Corresponding author affiliation and email:** Social Sciences Faculty, Department of Sociology, Universidad de Salamanca, Francisco Tomás y Valiente Avenue, no., 37071, Salamanca, Spain, macfadden@usal.es

SUPPLEMENTAL FILE 1

Example Search Strategies for Scopus

TITLE-ABS-KEY ((adolescent OR youth OR teen OR preadolescent OR child) AND ("culturally adapted intervention" OR "cultural adaptation" OR "cultural appropriateness" OR "program adaptation" OR "cross-cultural validation" OR "culturally tailored program" OR "cross-cultural adaptation" OR tailoring OR adaptation) AND ("evidence-based practice" OR "implementation science" OR "evidence-based intervention" OR "evidence-based case study" OR "preventive health care" OR prevention) AND (drug OR "substance abuse" OR "substance use prevention" OR "drug usage" OR alcohol OR "alcohol use" OR "underage drinking" OR "binge drinking" OR smoking OR "smoking prevention" OR "tobacco program" OR "tobacco smoking" OR "tobacco use" OR "cigarette smoking" OR vaping OR cannabis OR "cannabis use" OR "smoking blunts" "marijuana smoking" OR "marijuana abuse"))

TITLE-ABS-KEY ((adolescent OR youth OR teen OR preadolescent OR child) AND ("culturally adapted intervention" OR "cultural adaptation" OR "cultural appropriateness" OR "program adaptation" OR "cross-cultural validation" OR "culturally tailored program" OR "cross-cultural adaptation" OR tailoring OR adaptation) AND ("evidence-based practice" OR "implementation science" OR "evidence-based intervention" OR "evidence-based case study" OR "preventive health care" OR prevention) AND (anxiety OR "performance anxiety" OR depression OR "health promotion" OR "mental health promotion" OR "mental health program"))

Supplementary Table 1 Exact dates of the most recent searches conducted in each database

Last search date	Database
10/01/2024	PubMed
10/01/2024	Scopus
10/01/2024	PsycINFO
10/03/2024	Embase
10/03/2024	Web of Science
10/04/2024	CINAHL
10/04/2024	Cochrane
10/08/2024	OpenGrey
10/08/2024	WHO
10/08/2024	PAHO
10/08/2024	SAMHSA
10/12/2024	NIAAA
10/12/2024	NIDA
10/12/2024	EMCDDA
