# Supplementary material for: Exploring Cultural Adaptations: A Scoping Review on Adolescent Mental Health and Substance Use Prevention Programs
Source: Prev Sci. 2025 Jan 31;26(2):204–21. doi: 10.1007/s11121-025-01779-x (PMC11891097; doi:10.1007/s11121-025-01779-x)
Supplement: Supplementary file 2 — Supplementary file2 (PDF 289 KB) [file 11121_2025_1779_MOESM2_ESM.pdf]

Exploring Cultural Adaptations: A Scoping Review on Adolescent Mental Health and Substance Use Prevention Programs.
Prevention Science
Authors: Claudia Corpus-Espinosa, Isotta Mac Fadden*, María del Carmen Torrejón-Guirado, Marta Lima-Serrano
*** Corresponding author affiliation and email:** Social Sciences Faculty, Department of Sociology, Universidad de Salamanca, Francisco Tomás y Valiente Avenue, no., 37071, Salamanca, Spain, macfadden@usal.es

SUPPLEMENTAL FILE 2

References for programs included in the scoping review.

Abatemarco, D. J., West, B., Zec, V., Russo, A., Sosiak, P., & Mardesic, V. (2004). Project Northland in Croatia: A Community-Based Adolescent Alcohol Prevention Intervention. Journal Of Drug Education, 34(2), 167-178. https://doi.org/10.2190/hkln-exwb-8qpx-w8b8

Abreu, S., Murta, S. G., Rocha, V. P., & Pinheiro-Carozzo, N. (2021). *A Experiência Brasileira de Prevenção Escolar e Comunitária do Uso de Álcool e Outras Drogas: Registro Histórico de Adaptação, Implementação e Avaliação entre os Anos de 2013 a 2018*. *18*, 472–472. LILACS. https://doi.org/10.18310/9786587180526

Abuwalla, Z., Kadhem, Z., Gladstone, T., Mikhael, E., Bishay, A., & Van Voorhees, B. W. (2019). Proposed model for the cultural adaptation of an Internet-based depression prevention intervention (CATCH-IT) for Arab adolescents. *International Journal of Adolescent Medicine and Health*, *31*(1), 1–15. https://doi.org/10.1515/ijamh-2016-0147

Allen, D., Coombes, L., & Foxcroft, D. R. (2007). Cultural accommodation of the Strengthening Families Programme 10-14: UK Phase I study. *Health Education Research*, *22*(4), 547–560. https://doi.org/10.1093/her/cyl122

Amato, T. C., Opaleye, E. S., McBride, N., & Noto, A. R. (2021). Reducing alcohol-related risks among adolescents: A feasibility study of the shahrp program in Brazilian schools. *Ciencia e Saude Coletiva*, *26*(8), 3005–3018. Scopus. https://doi.org/10.1590/1413-81232021268.13472020

Authors: Claudia Corpus-Espinosa, Isotta Mac Fadden*, María del Carmen Torrejón-Guirado, Marta Lima-Serrano
*** Corresponding author affiliation and email:** Social Sciences Faculty, Department of Sociology, Universidad de Salamanca, Francisco Tomás y Valiente Avenue, no., 37071, Salamanca, Spain, macfadden@usal.es

Asdigian, N. L., Tuitt, N., Mousseau, A. C., Ivanich, J. D., Schultz, K., Keane, E. M., Zacher, T., Skinner, L., Richards, F. R. W., Bear Robe, L., & Whitesell, N. R. (2023). Grounding evidence-based prevention within cultural context: Promising effects of substance use prevention adapted for American Indian families. *Substance Use & Misuse*, *58*(8), 1004–1013. https://doi.org/10.1080/10826084.2023.2201847

Azziz-Baumgartner C, & Wilson, L. (2009). Applying a model of program adaptation to the Familias Fuertes parent/adolescent educational intervention for Latino immigrant families in the rural south. *Southern Online Journal of Nursing Research*, *9*(3), 7p–7p.

Baldus, C., Thomsen, M., Sack, P.-M., Bröning, S., Arnaud, N., Daubmann, A., & Thomasius, R. (2016). Evaluation of a German version of the Strengthening Families Programme 10-14: A randomised controlled trial. *European Journal of Public Health*, *26*(6), 953–959. https://doi.org/10.1093/eurpub/ckw082

Baldwin, J. A., Lowe, J., Brooks, J., Charbonneau-Dahlen, B. K., Lawrence, G., Johnson-Jennings, M., Padgett, G., Kelley, M., & Camplain, C. (2021). Formative Research and Cultural Tailoring of a Substance Abuse Prevention Program for American Indian Youth: Findings From the Intertribal Talking Circle Intervention. *Health Promotion Practice*, *22*(6), 778–785. https://doi.org/10.1177/1524839920918551

Bansa, M., Brown, D., DeFrino, D., Mahoney, N., Saulsberry, A., Marko-Holguin, M., Fogel, J., Gladstone, T. R. G., & Van Voorhees, B. W. (2018). A Little Effort Can Withstand the Hardship: Fielding an Internet-Based Intervention to Prevent Depression among Urban Racial/Ethnic Minority Adolescents in a Primary Care Setting. *Journal of the National Medical Association*, *110*(2), 130–142. https://doi.org/10.1016/j.jnma.2017.02.006

Authors: Claudia Corpus-Espinosa, Isotta Mac Fadden*, María del Carmen Torrejón-Guirado, Marta Lima-Serrano
*** Corresponding author affiliation and email:** Social Sciences Faculty, Department of Sociology, Universidad de Salamanca, Francisco Tomás y Valiente Avenue, no., 37071, Salamanca, Spain, macfadden@usal.es

Barrett, E. L., Newton, N. C., Teesson, M., Slade, T., & Conrod, P. J. (2015). Adapting the personality-targeted Preventure program to prevent substance use and associated harms among high-risk Australian adolescents. *Early Intervention in Psychiatry*, *9*(4), 308–315. Scopus. https://doi.org/10.1111/eip.12114

Bröning, S., Sack, P.-M., Thomsen, M., Stolle, M., Wendell, A., Stappenbeck, J., & Thomasius, R. (2014). Implementing and evaluating the German adaptation of the "Strengthening Families Program 10—14"- a randomized-controlled multicentre study. *BMC Public Health*, *14*, 83. https://doi.org/10.1186/1471-2458-14-83

Carney, T., Chimbambo, V., Johnson, K., Louw, J., & Myers, B. (2020a). The adaptation of an evidence-based brief intervention for substance-using adolescents and their caregivers. *Psychotherapy Research,* *30*(6), 728–738. https://doi.org/10.1080/10503307.2019.1656352

Carney, T., Johnson, K., Carrico, A., & Myers, B. (2020b). Acceptability and feasibility of a brief substance use intervention for adolescents in Cape Town, South Africa: A pilot study. *International Journal of Psychology,* *55*(6), 1016–1025. https://doi.org/10.1002/ijop.12668

Castro-Olivo, S. M., & Merrell, K. W. (2012). Validating cultural adaptations of a school-based social-emotional learning programme for use with Latino immigrant adolescents. *Advances in School Mental Health Promotion*, *5*(2), 78–92. https://doi.org/10.1080/1754730X.2012.689193

Clarke, A. T., Soto, G., Cook, J., Iloanusi, C., Akwarandu, A., & Parris, V. (2022). Adaptation of the Coping With Stress Course for Black Adolescents in Low-Income Communities: Examples of Surface Structure and Deep Structure Cultural Adaptations.

Authors: Claudia Corpus-Espinosa, Isotta Mac Fadden*, María del Carmen Torrejón-Guirado, Marta Lima-Serrano
*** Corresponding author affiliation and email:** Social Sciences Faculty, Department of Sociology, Universidad de Salamanca, Francisco Tomás y Valiente Avenue, no., 37071, Salamanca, Spain, macfadden@usal.es

Cognitive and Behavioral Practice, *29*(4), 738–749. https://doi.org/10.1016/j.cbpra.2021.04.005

Colby, M., Hecht, M., Miller-Day, M., Krieger, J., Syvertsen, A., Graham, J., & Pettigrew, J. (2013). Adapting School-Based Substance Use Prevention Curriculum Through Cultural Grounding: A Review and Exemplar of Adaptation Processes for Rural Schools. *American Journal of Community Psychology*, *51*(1/2), 190–205. https://doi.org/10.1007/s10464-012-9524-8

Cutrín, O., Kulis, S., Maneiro, L., MacFadden, I., Navas, M. P., Alarcón, D., Gómez-Fraguela, J. A., Villalba, C., & Marsiglia, F. F. (2021). Effectiveness of the Mantente REAL program for preventing alcohol use in Spanish adolescents. *Psychosocial Intervention, 30*(3), 113–122. https://doi.org/10.5093/PI2020A19

Cutrín, O., Fadden, I. M., Marsiglia, F. F., & Kulis, S. S. (2022). Social validity in Spain of the Mantente REAL prevention program for early adolescents: Social validity of Mantente REAL in Spain. *Journal of Prevention.* https://doi.org/10.1007/s10935-022-00701-3

De Castro-Amato, T. (2015). *Estudo de adaptação e viabilidade de um programa de redução de riscos do consumo de álcool para o contexto de escolas particulares no Brasil* [Universidaade Federal de São Paulo]. https://repositorio.unifesp.br/handle/11600/41674

De los Ángeles Luengo Martín, M., Tamames, E. R., Fraguela, J. A. G., López, A. G., & Pereiro, M. L. (1999). La prevención del consumo de drogas y la conducta antisocial en la escuela: análisis y evaluación de un programa. https://minerva.usc.es/xmlui/handle/10347/15497

Authors: Claudia Corpus-Espinosa, Isotta Mac Fadden*, María del Carmen Torrejón-Guirado, Marta Lima-Serrano
*** Corresponding author affiliation and email:** Social Sciences Faculty, Department of Sociology, Universidad de Salamanca, Francisco Tomás y Valiente Avenue, no., 37071, Salamanca, Spain, macfadden@usal.es

Debenham, J., Grummitt, L., Newton, N. C., Teesson, M., Slade, T., Conrod, P., & Kelly, E. V. (2021). Personality-targeted prevention for adolescent tobacco use: Three-year outcomes for a randomised trial in Australia. *Preventive Medicine*, *153*(106794), 106794. https://doi.org/10.1016/j.ypmed.2021.106794

Espada, J. P., Gonzálvez, M. T., Guillén-Riquelme, A., Sun, P., & Sussman, S. (2014). Immediate Effects of Project EX in Spain: A Classroom-Based Smoking Prevention and Cessation Intervention Program. *Journal of Drug Education*, *44*(1–2), 3–18. https://doi.org/10.1177/0047237915573523

Foxcroft, D. R., Callen, H., Davies, E. L., & Okulicz-Kozaryn, K. (2016). Effectiveness of the strengthening families programme 10–14 in Poland: cluster randomized controlled trial. European Journal Of Public Health, ckw195. https://doi.org/10.1093/eurpub/ckw195

Goldbach, J. T., & Holleran Steiker, L. K. (2011). An examination of cultural adaptations performed by LGBT-identified youths to a culturally grounded, evidence-based substance abuse intervention. *Journal of Gay & Lesbian Social Services: The Quarterly Journal of Community & Clinical Practice*, *23*(2), 188–203. https://doi.org/10.1080/10538720.2011.560135

Harthun, M. L., Dustman, P. A., Reeves, L. J., Marsiglia, F. F., & Hecht, M. L. (2009). Using Community-based Participatory Research to Adapt keepin' it REAL: Creating a Socially, Developmentally, and Academically Appropriate Prevention Curriculum for 5 Graders. *Journal of Alcohol and Drug Education*, *53*(3), 12–38.

Hecht, M. L., Elek, E., Wagstaff, D. A., Kam, J. A., Marsiglia, F., Dustman, P., Reeves, L., & Harthun, M. (2008). Immediate and short-term effects of the 5th grade version of

Authors: Claudia Corpus-Espinosa, Isotta Mac Fadden*, María del Carmen Torrejón-Guirado, Marta Lima-Serrano
*** Corresponding author affiliation and email:** Social Sciences Faculty, Department of Sociology, Universidad de Salamanca, Francisco Tomás y Valiente Avenue, no., 37071, Salamanca, Spain, macfadden@usal.es

the keepin' it REAL substance use prevention intervention. *Journal of Drug Education*, *38*(3), 225–251. https://doi.org/10.2190/DE.38.3.c

Hecht, M. L., Shin, Y., Pettigrew, J., Miller-Day, M., & Krieger, J. L. (2018). Designed Cultural Adaptation and Delivery Quality in Rural Substance Use Prevention: An Effectiveness Trial for the Keepin' it REAL Curriculum. *Prevention Science: The Official Journal of the Society for Prevention Research*, *19*(8), 1008–1018. https://doi.org/10.1007/s11121-018-0937-y

Holleran Steiker, L. K., Hopson, L. M., Goldbach, J. T., & Robinson, C. (2014). Evidence for Site-Specific, Systematic Adaptation of Substance Prevention Curriculum With High-Risk Youths in Community and Alternative School Settings. *Journal of Child & Adolescent Substance Abuse*, *23*(5), 307–317. https://doi.org/10.1080/1067828X.2013.869141

Ivanich, J. D., Mousseau, A. C., Walls, M., Whitbeck, L., & Whitesell, N. R. (2020). Pathways of Adaptation: Two Case Studies with One Evidence-Based Substance Use Prevention Program Tailored for Indigenous Youth. *Prevention Science: The Official Journal of the Society for Prevention Research*, *21*(Suppl 1), 43–53. https://doi.org/10.1007/s11121-018-0914-5

Jumper-Reeves, L., Dustman, P. A., Harthun, M. L., Kulis, S., & Brown, E. F. (2013). American Indian Cultures: How CBPR Illuminated Intertribal Cultural Elements Fundamental to an Adaptation Effort. Prevention Science, 15(4), 547-556. https://doi.org/10.1007/s11121-012-0361-7

Authors: Claudia Corpus-Espinosa, Isotta Mac Fadden*, María del Carmen Torrejón-Guirado, Marta Lima-Serrano
*** Corresponding author affiliation and email:** Social Sciences Faculty, Department of Sociology, Universidad de Salamanca, Francisco Tomás y Valiente Avenue, no., 37071, Salamanca, Spain, macfadden@usal.es

Komro, K. A. (2004). Brief Report: The Adaptation of Project Northland for Urban Youth. *Journal Of Pediatric Psychology*, *29*(6), 457-466. https://doi.org/10.1093/jpepsy/jsh049

Komro, K. A., Perry, C. L., Veblen-Mortenson, S., Farbakhsh, K., Kugler, K. C., Alfano, K. A., Dudovitz, B. S., Williams, C. L., & Jones-Webb, R. (2006). Cross-cultural adaptation and evaluation of a home-based program for alcohol use prevention among urban youth: The "Slick Tracy Home Team Program." *The Journal of Primary Prevention*, *27*(2), 135–154. https://doi.org/10.1007/s10935-005-0029-1

Komro, K. A., Perry, C. L., Veblen-Mortenson, S., Farbakhsh, K., Toomey, T. L., Stigler, M. H., Jones-Webb, R., Kugler, K. C., Pasch, K. E., & Williams, C. L. (2008). Outcomes from a randomized controlled trial of a multi-component alcohol use preventive intervention for urban youth: Project Northland Chicago. *Addiction*, *103*(4), 606-618. https://doi.org/10.1111/j.1360-0443.2007.02110.x

Kulis, S. S., Ayers, S. L., & Harthun, M. L. (2016). Substance Use Prevention for Urban American Indian Youth: A Efficacy Trial of the Culturally Adapted Living in 2 Worlds Program. The Journal Of Primary Prevention, 38(1-2), 137-158. https://doi.org/10.1007/s10935-016-0461-4

Kulis, S. S., Marsiglia, F. F., Medina-Mora, M. E., Nuño-Gutiérrez, B. L., Corona, M. D., & Ayers, S. L. (2021). Keepin' It REAL-Mantente REAL in Mexico: A Cluster Randomized Controlled Trial of a Culturally Adapted Substance Use Prevention Curriculum for Early Adolescents. *Prevention Science*, *22*(5), 645–657. https://doi.org/10.1007/s11121-021-01217-8

Authors: Claudia Corpus-Espinosa, Isotta Mac Fadden[*], María del Carmen Torrejón-Guirado, Marta Lima-Serrano
*** Corresponding author affiliation and email:** Social Sciences Faculty, Department of Sociology, Universidad de Salamanca, Francisco Tomás y Valiente Avenue, no., 37071, Salamanca, Spain, macfadden@usal.es

Kumpfer, K., Xie, J., & O'Driscoll, R. (2012). Effectiveness of a Culturally Adapted Strengthening Families Program 12-16 Years for High-Risk Irish Families. *Child & Youth Care Forum*, *41*(2), 173–195. https://doi.org/10.1007/s10566-011-9168-0

Kyritsi, K. P., & Bacopoulou, F. (2021). Cultural Accommodation of the Strengthening Families Program for Parents and Young Adolescents 10–14: Greek Phase I and II Study. *Advances in Experimental Medicine and Biology*, *1339*, 283–299. Scopus. https://doi.org/10.1007/978-3-030-78787-5_34

Lima-Serrano, M., Martínez-Montilla, J. M., Lima-Rodríguez, J. S., Mercken, L., & De Vries, H. (2018). Design, implementation, and evaluation of a web-based computer-tailored intervention to prevent binge drinking in adolescents: Study protocol. *BMC Public Health, 18*(1), Article 5346. https://doi.org/10.1186/s12889-018-5346-4

Lowe, J., Brooks, J., Lawrence, G., Baldwin, J. A., Kelley, M., & Wimbish-Tompkins, R. (2024). Intertribal Talking Circle for the prevention of alcohol and drug use among Native American youth. *Research In Nursing & Health*, *47*(2), 234-241. https://doi.org/10.1002/nur.22372

Marsiglia, F. F., Kulis, S. S., Cutrín, O., Medina-Mora, M. E., Real, T., Nuño-Gutiérrez, B. L., Corona, M. D., Mendoza-Meléndez, M. Á., Gresenz, K., & Alcala-Calvillo, D. (2022). The Feasibility, Acceptability, and Utility of Mantente REAL: The Culturally Adapted Version of keepin' it REAL for Mexico. *Prevention Science: The Official Journal of the Society for Prevention Research*, *23*(8), 1483–1494. https://doi.org/10.1007/s11121-022-01409-w

Marsiglia, F. F., Medina-Mora, M. E., Gonzalvez, A., Alderson, G., Harthun, M., Ayers, S., Gutiérrez, B. N., Corona, M. D., Melendez, M. A. M., & Kulis, S. (2019). Binational

Authors: Claudia Corpus-Espinosa, Isotta Mac Fadden*, María del Carmen Torrejón-Guirado, Marta Lima-Serrano
*** Corresponding author affiliation and email:** Social Sciences Faculty, Department of Sociology, Universidad de Salamanca, Francisco Tomás y Valiente Avenue, no., 37071, Salamanca, Spain, macfadden@usal.es

Cultural Adaptation of the keepin' it REAL Substance Use Prevention Program for Adolescents in Mexico. *Prevention Science: The Official Journal of the Society for Prevention Research*, *20*(7), 1125–1135. https://doi.org/10.1007/s11121-019-01034-0

Martínez-Montilla, J. M. (2020). *Alerta Alcohol. Design and evaluation of a web-based computer-tailored intervention for prevention of alcohol consumption and binge drinking in Spanish adolescents.* https://idus.us.es/handle/11441/103462

Martínez-Montilla, J. M., Mercken, L., De Vries, H., Candel, M., Lima-Rodríguez, J. S., & Lima-Serrano, M. (2020). A web-based, computer-tailored intervention to reduce alcohol consumption and binge drinking among Spanish adolescents: Cluster randomized controlled trial. *Journal of Medical Internet Research, 22*(1), Article e15438. https://doi.org/10.2196/15438

Medeiros, P. F. P., Cruz, J. I., R Schneider, D., Sanudo, A., & Sanchez, Z. M. (2016). Process evaluation of the implementation of the Unplugged Program for drug use prevention in Brazilian schools. *Substance Abuse Treatment, Prevention, and Policy*, *11*, 2. https://doi.org/10.1186/s13011-015-0047-9

Montero-Zamora, P., Brown, E. C., Ringwalt, C. L., Schwartz, S. J., Prado, G., & Ortiz-García, J. (2021a). Predictors of engagement and attendance of a family-based prevention program for underage drinking in Mexico. *Prevention Science, 23*(2), 237–247. https://doi.org/10.1007/s11121-021-01301-z

Montero-Zamora, P., St. Fleur, R. G., Mejía-Trujillo, J., & Brown, E. C. (2021b). Contextual fit of a family evidence-based intervention for preventing youth alcohol use in Mexico. *Journal of Primary Prevention, 42*(5), 441–457. https://doi.org/10.1007/s10935-021-00640-5

Authors: Claudia Corpus-Espinosa, Isotta Mac Fadden*, María del Carmen Torrejón-Guirado, Marta Lima-Serrano
*** Corresponding author affiliation and email:** Social Sciences Faculty, Department of Sociology, Universidad de Salamanca, Francisco Tomás y Valiente Avenue, no., 37071, Salamanca, Spain, macfadden@usal.es

Montero-Zamora, P., Brown, E. C., Ringwalt, C. L., Schwartz, S. J., Prado, G., & Ortiz-García, J. (2023). Effects of a family-based program for reducing risk for youth alcohol use in Mexico. *Journal of Child and Family Studies, 33*(4), 1224–1237. https://doi.org/10.1007/s10826-023-02725-7

Murta, S. G., de Almeida Nobre-Sandoval, L., Rocha, V. P. S., Miranda, A. A. V., Duailibe, K. D., Farias, D. A., de Menezes, J. C. L., Abdala, I. G., do Socorro Mendes Gomes, M., & do Amaral Vinha, L. G. (2021). Social Validity of the Strengthening Families Program in Northeastern Brazil: The Voices of Parents, Adolescents, and Facilitators. *Prevention Science: The Official Journal of the Society for Prevention Research*, *22*(5), 658–669. https://doi.org/10.1007/s11121-020-01173-9

Murta, S. G., nueva, E. a sitio externo E. enlace se abrirá en una ventana, Nobre-Sandoval, L. de A., Pedralho, M. de S., Tavares, T. N. G., Ramos, C. E. P. L., Allen, D., & Coombes, L. (2018). Needs assessment for cultural adaptation of Strengthening Families Program (SFP 10-14-UK) in Brazil. *Psicologia, Reflexão e Crítica*, *31*(1), 1–12. https://doi.org/10.1186/s41155-018-0105-0

Murta, S. G., Vinha, L. G. do A., Nobre-Sandoval, L. de A., Miranda, A. A. V., Menezes, J. C. L. de, & Rocha, V. P. S. (2020). Feasibility of the Strengthening Families Program for Brazilian Families: A Mixed Method Study. *Psicologia: Teoria e Pesquisa*, *36*, e36nspe16. https://doi.org/10.1590/0102.3772e36nspe16

Noël, L. T. (2014). A Randomized Controlled Trial of a Depression Prevention Curriculum for Rural Middle School Girls: Initial Findings and 6-Month Follow-up. *Journal of Child and Adolescent Behaviour*, *02*(02). https://doi.org/10.4172/2375-4494.1000127

Authors: Claudia Corpus-Espinosa, Isotta Mac Fadden*, María del Carmen Torrejón-Guirado, Marta Lima-Serrano
*** Corresponding author affiliation and email:** Social Sciences Faculty, Department of Sociology, Universidad de Salamanca, Francisco Tomás y Valiente Avenue, no., 37071, Salamanca, Spain, macfadden@usal.es

Noël, L. T., Rost, K., & Gromer, J. (2013). A Depression Prevention Program for Rural Adolescents: Modification and Design. *Children & Schools*, *35*(4), 199–211. https://doi.org/10.1093/cs/cdt018

Okulicz-Kozaryn, K., & Dorozko, L. (2008). A Polish adaptation of the SFP alcohol prevention program for 10–14-year-olds and their parents [In Polish]. In K. Okulicz-Kozaryn & K. Ostaszewski (Eds.), Promocja zdrowia psychicznego—badania i dzialania w Polsce (pp. 249–262). Instytut Psychiatrii i Neurologii.

Orpinas, P., Reidy, M. C., Lacy, M. E., Kogan, S. M., Londoño-McConnell, A., & Powell, G. (2014). Familias fuertes: A feasibility study with Mexican immigrants living in low-income conditions in the southeastern United States. *Health Promotion Practice*, *15*(6), 915–923. https://doi.org/10.1177/1524839914523430

Ortega, E., Giannotta, F., Latina, D., & Ciairano, S. (2012). Cultural Adaptation of the Strengthening Families Program 10–14 to Italian Families. *Child & Youth Care Forum*, *41*(2), 197–212. https://doi.org/10.1007/s10566-011-9170-6

Patchell, B. A. (2011). Native American Indian adolescents: Response to a culturally tailored, school -based substance abuse intervention. In *Native American Indian Adolescents: Response to a Culturally Tailored, School -based Substance Abuse Intervention* (UMI Order AAI3534162; p. 186 p). New Mexico State University.

Pedroso, R. T., & Hamann, E. M. (2019). Adaptations of the pilot of the Unplugged#tamojunto program for health promotion and drug prevention in Brazilian schools. *Ciencia & Saude Coletiva*, *24*(2), 371–381. https://doi.org/10.1590/1413-81232018242.32932016

Authors: Claudia Corpus-Espinosa, Isotta Mac Fadden*, María del Carmen Torrejón-Guirado, Marta Lima-Serrano
*** Corresponding author affiliation and email:** Social Sciences Faculty, Department of Sociology, Universidad de Salamanca, Francisco Tomás y Valiente Avenue, no., 37071, Salamanca, Spain, macfadden@usal.es

Pérez, J. M. E., Díaz, S. A.-H., Villa, R. S., Fernández-Hermida, J. R., Carballo, J. L., & García-Rodríguez, O. (2010). Family-based drug use prevention: The “Familias que Funcionan” program. *Psychology in Spain*, *14*(1), 1–7. APA PsycInfo®.

Roehrig, C., & Pradier, C. (2017). Strengthening Families Program: Key points for the French adaptation. *Sante Publique*, *29*(5), 643–653. Scopus. https://doi.org/10.3917/spub.175.0643

Sáez-Santiago, E., Rodríguez-Hernández, N., Núñez-Méndez, A., & Bernal, G. (2017). Cultural adaptation of a school-based depression prevention program for adolescents in Puerto Rico. In E. C. [Ed Lopez, S. G. [Ed Nahari, & S. L. [Ed Proctor (Eds.), *Handbook of multicultural school psychology: An interdisciplinary perspective (2nd ed.)* (pp. 172–189, 466 Pages). Routledge/Taylor & Francis Group (New York, NY, US). https://doi.org/10.4324/9780203754948-10

Sanchez, Z. M., Valente, J. Y., Galvão, P. P., Gubert, F. A., Melo, M. H. S., Caetano, S. C., Mari, J. J., & Cogo-Moreira, H. (2021). A cluster randomized controlled trial evaluating the effectiveness of the school-based drug prevention program #Tamojunto2.0. *Addiction*, *116*(6), 1580–1592. https://doi.org/10.1111/add.15358

Sanchez, Z. M., Valente, J. Y., Gubert, F. A., Galvão, P. P. O., Cogo-Moreira, H., Rebouças, L. N., dos Santos, M. H. S., Melo, M. H. S., & Caetano, S. C. (2024). Short-term effects of the strengthening families Program (SFP 10–14) in Brazil: a cluster randomized controlled trial. *Child and Adolescent Psychiatry and Mental Health*, *18*(1). https://doi.org/10.1186/s13034-024-00748-6

Sánchez-Franco, S., Arias, L. F., Jaramillo, J., Murray, J. M., Hunter, R. F., Llorente, B., Bauld, L., Good, S., West, J., Kee, F., & Sarmiento, O. L. (2021). Cultural adaptation of

Authors: Claudia Corpus-Espinosa, Isotta Mac Fadden*, María del Carmen Torrejón-Guirado, Marta Lima-Serrano
*** Corresponding author affiliation and email:** Social Sciences Faculty, Department of Sociology, Universidad de Salamanca, Francisco Tomás y Valiente Avenue, no., 37071, Salamanca, Spain, macfadden@usal.es

two school-based smoking prevention programs in Bogotá, Colombia. *Translational Behavioral Medicine*, *11*(8), 1567–1578. https://doi.org/10.1093/tbm/ibab019

Saulsberry, A., Corden, M. E., Taylor-Crawford, K., Crawford, T. J., Johnson, M., Froemel, J., Walls, A., Fogel, J., Marko-Holguin, M., & van Voorhees, B. W. (2013). Chicago Urban Resiliency Building (CURB): An Internet-Based Depression-Prevention Intervention for Urban African-American and Latino Adolescents. *Journal of Child and Family Studies*, *22*(1), 150–160. Scopus. https://doi.org/10.1007/s10826-012-9627-8

Segrott, J., Gillespie, D., Lau, M., Holliday, J., Murphy, S., Foxcroft, D., Hood, K., Scourfield, J., Phillips, C., Roberts, Z., Rothwell, H., Hurlow, C., & Moore, L. (2022). Effectiveness of the Strengthening Families Programme in the UK at preventing substance misuse in 10–14 year-olds: A pragmatic randomised controlled trial. *BMJ Open*, *12*(2), e049647. https://doi.org/10.1136/bmjopen-2021-049647

Skärstrand, E., Larsson, J., & Andréasson, S. (2008). Cultural adaptation of the Strengthening Families Programme to a Swedish setting. *Health Education*, *108*(4), 287–300. https://doi.org/10.1108/09654280810884179

Skärstrand, E., Sundell, K., & Andréasson, S. (2014). Evaluation of a Swedish version of the Strengthening Families Programme. *European Journal of Public Health*, *24*(4), 578–584. https://doi.org/10.1093/eurpub/ckt146

Stolle, M., Stappenbeck, J., Wendell, A., & Thomasius, R. (2011). Family-based prevention against substance abuse and behavioral problems: Culture-sensitive adaptation process for the modification of the US-American Strengthening Families Program 10-14 to German conditions. *Journal of Public Health (09431853)*, *19*(4), 389–395. https://doi.org/10.1007/s10389-011-0405-7

Authors: Claudia Corpus-Espinosa, Isotta Mac Fadden*, María del Carmen Torrejón-Guirado, Marta Lima-Serrano
*** Corresponding author affiliation and email:** Social Sciences Faculty, Department of Sociology, Universidad de Salamanca, Francisco Tomás y Valiente Avenue, no., 37071, Salamanca, Spain, macfadden@usal.es

Tamí-Maury, I., Noé-Díaz, V., García, H., Amell, L., Betancur, A., Chen, M., Pérez, O., Calabro, K. S., Yepes, A., Ríos, X., Sansores, R., Prokhorov, A. V., Tamí-Maury, I., Noé-Díaz, V., García, H., Amell, L., Betancur, A., Chen, M., Pérez, O., … Prokhorov, A. V. (2019). Adapting a computer-based smoking prevention program to Latin American adolescents. *Neumología y Cirugía de Tórax*, *78*(4), 342–347. https://doi.org/10.35366/NT194B

Vargas-Martínez, A. M., Lima-Serrano, M., & Trapero-Bertrán, M. (2023). Cost-effectiveness and cost-utility analyses of a web-based computer-tailored intervention for prevention of binge drinking among Spanish adolescents. *Alcoholism: Clinical and Experimental Research*. https://doi.org/10.1111/acer.14990

Velasco, V., Griffin, K. W., Antichi, M., & Celata, C. (2015). A large-scale initiative to disseminate an evidence-based drug abuse prevention program in Italy: Lessons learned for practitioners and researchers. *Evaluation and Program Planning*, *52*, 27–38. https://doi.org/10.1016/j.evalprogplan.2015.03.002

Velasco, V., Griffin, K. W., & Botvin, G. J. (2017). Preventing Adolescent Substance Use Through an Evidence-Based Program: Effects of the Italian Adaptation of Life Skills Training. *Prevention Science : The Official Journal of the Society for Prevention Research*, *18*(4), 394–405. Medline. https://doi.org/10.1007/s11121-017-0776-2

West, B., Abatemarco, D., Ohman-Strickland, P. A., Zec, V., Russo, A., & Milic, R. (2008). Project Northland in Croatia: Results and Lessons Learned. Journal Of Drug Education, 38(1), 55-70. https://doi.org/10.2190/de.38.1.e

Whitesell, N. R., Mousseau, A. C., Keane, E. M., Asdigian, N. L., Tuitt, N., Morse, B., Zacher, T., Dick, R., Mitchell, C. M., & Kaufman, C. E. (2019). Integrating Community-

Authors: Claudia Corpus-Espinosa, Isotta Mac Fadden*, María del Carmen Torrejón-Guirado, Marta Lima-Serrano
*** Corresponding author affiliation and email:** Social Sciences Faculty, Department of Sociology, Universidad de Salamanca, Francisco Tomás y Valiente Avenue, no., 37071, Salamanca, Spain, macfadden@usal.es

Engagement and a Multiphase Optimization Strategy Framework: Adapting Substance Use Prevention for American Indian Families. *Prevention Science*, *20*(7), 1136–1146. Scopus. https://doi.org/10.1007/s11121-019-01036-y

Wimbish-Cirilo, R. M. (2016). *Culturally tailoring a substance use intervention among south eastern urban American Indian and Alaska Native youth*. Florida Atlantic University.
