# Supplementary material for: Exploring Cultural Adaptations: A Scoping Review on Adolescent Mental Health and Substance Use Prevention Programs
Source: Prev Sci. 2025 Jan 31;26(2):204–21. doi: 10.1007/s11121-025-01779-x (PMC11891097; doi:10.1007/s11121-025-01779-x)
Supplement: Supplementary file 3 — Supplementary file3 (PDF 189 KB) [file 11121_2025_1779_MOESM3_ESM.pdf]

Exploring Cultural Adaptations: A Scoping Review on Adolescent Mental Health and Substance Use Prevention Programs.
Prevention Science
Authors: Claudia Corpus-Espinosa, Isotta Mac Fadden*, María del Carmen Torrejón-Guirado, Marta Lima-Serrano
*** Corresponding author affiliation and email:** Social Sciences Faculty, Department of Sociology, Universidad de Salamanca, Francisco Tomás y Valiente Avenue, no., 37071, Salamanca, Spain, macfadden@usal.es

SUPPLEMENTAL FILE 3

Supplementary Table 2 Characteristics of the programs

Adapted intervention and origin	Prevention objective; context; adaptation country; theory; intervention period; facilitator; evaluation design.
Alerta Alcohol, Netherlands (Author, year; Author, year; Martínez-Montilla, 2020; Author,year).	To decrease the alcohol consumption and binge drinking in adolescents; School-based; Spain; I-Change model; Six sessions, spaced one to two weeks apart; Web-based (self-administered); Descriptive study, Pilot study, cluster RCT, Economic study.
ASPIRE, USA (Tamí-Maury et al., 2019).	To prevent and to cease smoking; School-based; Colombia and Mexico; Transtheoretical Model of Change and the PRECEDE-PROCEED model; 5 modules; An animated older peer (computer-based); Multy-country pilot study.
Bacanísimo, UK (Sánchez-Franco et al., 2021).	To prevent smoking onset among students; School-based; Bogota; Personal skills learning, theory of planned behavior and conventional classroom pedagogy; 9 sessions; Trained teachers; Concurrent nested mixed methods study.
Project Competent Adulthood Transition with Cognitive-behavioral, Humanistic and Interpersonal Training (CATCH-IT), USA (Abuwalla et al., 2019).	To build on the youth resiliency and to prevent depression among at-risk adolescents; School-based; Arab Countries; Behavioral activation, cognitive-behavioral therapy (CBT), interpersonal psychotherapy, community resiliency concept model; 14 modules; Web-based (self-administered); Qualitative study.
Chicago Urban Resiliency Building (CURB), USA (Bansa et al., 2018; Saulsberry et al., 2013).	To build on the youth resiliency and to prevent depression among at-risk adolescents; Community-based; USA (Chicago); Behavioral activation, cognitive-behavioral therapy (CBT), interpersonal psychotherapy, community resiliency concept model; 14 modules; Web-based (self-administered); Descriptive study, mix-method.
Entrenamiento en Habilidades de Vida (De los Ángeles Luengo Martín et al., 1999)	To reduce alcohol, tobacco, drug abuse and violence; School-based; Spain; Social learning theory, problem behavior theory, self-derogation, persuasive communications, and peer cluster theories; 17 sessions (50 min each); Trained teachers; Descriptive study, quasi-experimental design
Entre Parceros, UK. (Sánchez-Franco et al., 2021)	To prevent smoking onset among students; School-based; Bogota; Social influence approach; 2-day course; Trained peer supporters; Concurrent nested mixed methods study.
Estrategias para mantener un ánimo saludable (EMAS), Puerto Rico	To reduce risk factors while strengthening the protective factors that may prevent the development of depression among adolescents; School-based; Puerto Rico; Cognitive

(Sáez-Santiago et al., 2017).	behavioral therapy; 14 weekly sessions (40-50 mins); Teachers; Descriptive study.
Familias Fuertes (FF), UK (Abreu et al., 2021; Murta et al., 2018, 2020, 2021; Sanchez et al., 2024).	To prevent or to delay the onset of substance use and other problem behaviors as well as to reinforce parenting skills and strengthen the family; Family-based; Brazil; The theory of family systems, social cognition theory, the model of resilience, and socio-ecological model; 7 sessions (2 hrs.) plus 4 optional booster sessions; Social educators, federal multipliers, psychologists, social workers, historians; Qualitative study, a descriptive study, mixed-methods study, qualitative study, Cluster RCT
Familias Fuertes (FF), USA (Azziz-Baumgartner C & Wilson, 2009; Orpinas et al., 2014).	To prevent or to delay the onset of substance use and other problem behaviors as well as to reinforce parenting skills and strengthen the family; Family-based; USA (Latino immigrant families in the rural South); The theory of family systems, social cognition theory, the model of resilience, and socio-ecological model; 7 sessions (2 hrs.) plus 4 optional booster sessions ; Latino community volunteers; Descriptive study, exploratory design.
Familias que funcionan, USA (Pérez et al., 2010).	To prevent or to delay the onset of substance use and other problem behaviors as well as to reinforce parenting skills and strengthen the family; Family-based; Spain; The theory of family systems, social cognition theory, the model of resilience, and socio-ecological model; 7 sessions (2 hrs.) plus 4 optional booster sessions; Trained facilitators; Longitudinal study.
Familien stärken, USA (Baldus et al., 2016; Bröning et al., 2014; Stolle et al., 2011).	To prevent or to delay the onset of substance use and other problem behaviors as well as to reinforce parenting skills and strengthen the family; Family-based; Germany; The theory of family systems, social cognition theory, the model of resilience, and socio-ecological model; 7 sessions (2 hrs.) plus 4 optional booster sessions; Trained facilitators; Pilot study, multi-centered RCT.
Guiding Good Choices, USA (Montero-Zamora et al., 2021a, 2021b, 2022)	To address risk and protective factors for preventing underage drinking through parenting practices; Family-based; Mexico; Social Development Model; 5 sessions; (2hrs.); A certified trainer; Qualitative study; quasi-experimental
Intertribal Talking Circle (ITC) USA (Baldwin et al., 2021, Lowe, 2024)	To prevent alcohol use and drug use; Community-based; USA (tree tribes within USA); Native reliance theoretical model; 10 weekly sessions (30 mins.); Adult facilitator; Descriptive study; quasi-experimental study.
Jóvenes Fuertes, USA (Castro-Olivo & Merrell, 2012).	To promote SEL resiliency skills to prevent depression and anxiety; School-based; USA (Latino immigrant population); Social-emotional learning; 12 lessons;

	Bilingual and bicultural certified teachers; Pretest-posttest without true control group.
Keepin´it REAL (KiR), USA (Goldbach & Holleran Steiker, 2011).	To reduce alcohol, tobacco and marijuana use and increasing anti-drugs attitudes as well as, to teach a variety of communication techniques to navigate risky or undesirable influences; Community-based; USA (LGBT+ community); Communication competence theory, ecological risk, and resiliency approach; Not mentioned; Not mentioned; Qualitative study.
Keepin´it REAL (KiR), USA (Colby et al., 2013; Hecht et al., 2018).	To reduce alcohol, tobacco and marijuana use and increasing anti-drugs attitudes as well as, to teach a variety of communication techniques to navigate risky or undesirable influences; School-based; USA (Pennsylvania and Ohio rural Communities); Communication competence theory, the ecological risk and resiliency approach; 12 sessions (45 mins); Teachers; Descriptive study, RCT.
Keepin´it REAL (KiR) USA (Holleran Steiker et al., 2014).	To reduce alcohol, tobacco and marijuana use and increasing anti-drugs attitudes as well as, to teach a variety of communication techniques to navigate risky or undesirable influences; School-based; USA (Texas); Communication competence theory, the ecological risk and resiliency approach; 6 weekly sessions (60-90 mins.); Not mentioned; Quasi-experimental design and focus groups.
Keepin´it REAL (KiR), USA (Harthun et al., 2009; Hecht et al., 2008).	To reduce alcohol, tobacco and marijuana use and increasing anti-drugs attitudes as well as, to teach a variety of communication techniques to navigate risky or undesirable influences; School-based; USA (5h grade); Communication competence theory, the ecological risk and resiliency approach; 12 sessions; Teachers; CBPR study, longitudinal study.
Life Skills Training Program (LST), USA (Velasco et al., 2015, 2017).	To reduce alcohol, tobacco, drug abuse and violence; School-based; Italy; Social learning theory, problem behavior theory, self-derogation, persuasive communications, and peer cluster theories; Three levels, one per year. First year: 15 sessions. Second year: 10 sessions. Third year: 9 sessions; Trained teachers; Descriptive study, quasi-experimental design with control group.
Living in two Worlds (L2W) (Jumper-Reeves et al., 2013; Kulis et al., 2016)	To reduce alcohol, tobacco and marijuana use and increasing anti-drugs attitudes as well as, to teach a variety of communication techniques to navigate risky or undesirable influences; School-based; USA (Urban American Indian youth); Communication competence theory, the ecological risk and resiliency approach; 12 sessions (45 mins); Teachers; Qualitative study, RCT

Mantente REAL (MREAL), USA (Kulis et al., 2021; Marsiglia et al., 2019, 2022).	To reduce alcohol, tobacco and marijuana use and increasing anti-drugs attitudes as well as, to teach a variety of communication techniques to navigate risky or undesirable influences; School-based; Mexico; Communication competence theory, the ecological risk and resiliency approach; 12 sessions (45 mins); Teachers; Descriptive study, cluster RCT, mix-method study.
Mantente REAL (MREAL), USA (Author, year; Author, year)	To reduce alcohol, tobacco and marijuana use and increasing anti-drugs attitudes as well as, to teach a variety of communication techniques to navigate risky or undesirable influences; School-based; Spain; Communication competence theory, the ecological risk and resiliency approach; 12 sessions (45 mins); Tutors (teachers); Small RCT, mixed-method design.
Native Talking Circle (NTC) USA (Patchell, 2011).	To prevent alcohol use and drug use; School-based; USA (Native American Indian tribal groups in rural Oklahoma); Native self-reliance model; 10 sessions (30-45 mins) 2-3 times per week for 8.5 weeks; female non-tribal member; Exploratory quantitative study.
PERAE (Programa de Estímulo à Saúde e Redução de Riscos Associados ao Uso de Álcool Aplicado ao Ambiente Educacional), Australia (Amato et al., 2021; De Castro-Amato, 2015).	To reduce drinking and alcohol-related harm among adolescents; School-based; Brazil; Inoculation theory, harm reduction approach; 15 sessions (40-60 mins.); Trained teachers; Mixed-method study.
Preventure, UK (Barrett et al., 2015; Debenham et al., 2021).	To address unique personality specific motivations for risky drinking; School-based; Australia; Psychoeducational, cognitive-behavioral, and motivational-interviewing components; 2 sessions; Trained facilitators; Qualitative study, cluster RCT
Project Ex, USA (Espada et al., 2014).	To prevent or cease tobacco use among adolescents; School-based; Spain; Not mentioned;<br>8 sessions in 6 weeks; Psychology graduate students; RCT.
Project Northland, USA (Komro, 2004; Komro et al., 2008)	To prevent early-onset alcohol use among adolescents; Multiple-component (school-based, family-based and community-based); USA (Chicago); The social influences theory of behavioral change; 3-year intervention; Teachers and trained project staff; Descriptive study, RCT
Project Northland Croatia, USA (Abatemarco et al., 2004; West et al., 2008)	To prevent early-onset alcohol use among adolescents; Multiple-component (school-based, family-based and community-based); Croatia; The social influences theory of behavioral change; 3-year intervention; Teachers and a project coordinator; mix-method study, RCT

RAD-PAL, USA Carney et al., 2020a, 2020b)	To prevent substance use and other risk behaviors; Family-based; South Africa; Cognitive behavioral therapy and motivational interviewing; 3 sessions; Trained staff; Descriptive study. Mix-methos study
Resilient In spite of Stressful Events (RISE), USA (Clarke et al., 2022).	To prevent depression among adolescents at increased risk for depression due to known risk factors; Community-based; USA (black adolescents from low-income urban communities); Transactional model of stress and coping, the family process model of economic hardship and child adjustment and the adaptation to poverty-related stress model; 9 weekly sessions (105 mins.); Trained graduate student co-leaders and undergraduate student program assistants, under the supervision of a licensed psychologist; Descriptive study.
Strengthening families program (SFP), USA (Roehrig & Pradier, 2017).	To prevent or to delay the onset of substance use and other problem behaviors as well as to reinforce parenting skills and strengthen the family; Family-based; France; The theory of family systems, social cognition theory, the model of resilience, and socio-ecological model; 14 sessions (2 hrs.); Trained municipal services professionals; Pilot study.
Strengthening families program (10-14), UK (Kyritsi & Bacopoulou, 2021).	To prevent or to delay the onset of substance use and other problem behaviors as well as to reinforce parenting skills and strengthen the family; Family-based; Greece; The theory of family systems, social cognition theory, the model of resilience, and socio-ecological model; 7 sessions (2 hrs.) plus 4 optional booster sessions; Certified facilitators; Pilot study.
Strengthening families program (10-14), USA (Ortega et al., 2012).	To prevent or to delay the onset of substance use and other problem behaviors as well as to reinforce parenting skills and strengthen the family; Family-based; Italy; The theory of family systems, social cognition theory, the model of resilience, and socio-ecological model; 7 sessions (2 hrs.) plus 4 optional booster sessions; Certified facilitators; Pilot study.
Strengthening families program (10-14), UK (Foxcroft et al., 2016; Okulicz-Kozaryn & Dorozko, 2008)	To prevent or to delay the onset of substance use and other problem behaviors as well as to reinforce parenting skills and strengthen the family; Family-based; Poland; The theory of family systems, social cognition theory, the model of resilience, and socio-ecological model; 7 sessions (2 hrs.) plus 4 optional booster sessions; Certified facilitators; Descriptive study, Cluster RCT.
Strengthening families program (10-14), USA (Allen et al., 2007; Segrott et al., 2022).	To prevent or to delay the onset of substance use and other problem behaviors as well as to reinforce parenting skills and strengthen the family; Family-based; UK; The theory of family systems, social cognition theory, the model of

	resilience, and socio-ecological model; 7 sessions (2 hrs.) plus 4 optional booster sessions; Certified facilitators; Qualitative study, pragmatic cluster-RCT.
Strengthening families program (SFP 10-14), USA (Skärstrand et al., 2008, 2014).	To prevent or to delay the onset of substance use and other problem behaviors as well as to reinforce parenting skills and strengthen the family; School-based; Sweden; The theory of family systems, social cognition theory, the model of resilience, and socio-ecological model; 12 sessions (1:30-2 hrs.); Teachers and group leaders; Descriptive study, cluster RCT.
Strengthening families program 12-16 for at-risk families (SFP 12-16), USA (Kumpfer et al., 2012).	To prevent or to delay the onset of substance use and other problem behaviors as well as to reinforce parenting skills and strengthen the family; Family-based; Ireland; The theory of family systems, social cognition theory, the model of resilience, and socio-ecological model; 14 sessions; Professionals from a variety of disciplines; Quasi-experimental design.
Slick Tracy Home Team Program, USA (Komro et al., 2006).	To prevent early-onset alcohol use among young adolescents; School-based; USA (Chicago); The theory of triadic and Perry's planning model for adolescent health promotion programs; 11 weekly sessions; Teachers and peer leaders; RCT.
TALKnTIME, USA. (Noël, 2014; Noël et al., 2013).	To cope with future episodes of stress and sadness to prevent full onset of major depression; School-based; USA (rural community); Cognitive-behavioral principles and Positive Youth development principles; 12 weekly sessions (90 min.); High-school facilitator; Mix-method study (pilot study), RCT.
Thiwáhe Gluwáš'akapi (TG), USA (Asdigian et al., 2023; Ivanich et al., 2020; Whitesell et al., 2019).	To prevent or to delay the onset of substance use and other problem behaviors as well as to reinforce parenting skills and strengthen the family; Family-based; USA (northern plains tribe); The theory of family systems, social cognition theory, the model of resilience, and socio-ecological model; 7 sessions (2 hrs.) plus 4 optional booster sessions; Trained community members; Case study, Pilot study, Pretest-posttest design (short-term effects).
#Tamojunto, Europe (Medeiros et al., 2016; Pedroso & Hamann, 2019; Sanchez et al., 2021).	To prevent drug misuse in the school environment; School-based; Brazil; Comprehensive social influence approach and combines teaching life skills and normative contents; 12 sessions weekly (40-50mins.); Teachers; Qualitative study, qualitative study, cluster RCT.
Urban Talking Circle (UTC), USA (Cherokee community) (Wimbish-Cirilo, 2016).	To prevent alcohol use and drug use; Community-based; USA (urban American Indian/ Alaskan native community); Native reliance theoretical model, theory of nursing as caring; 10 weekly sessions (30 mins.); A trained person

	and an urban American Indian nurse; 2-condition quasi-experimental design.
