# Supplementary material for: Exploring Cultural Adaptations: A Scoping Review on Adolescent Mental Health and Substance Use Prevention Programs
Source: Prev Sci. 2025 Jan 31;26(2):204–21. doi: 10.1007/s11121-025-01779-x (PMC11891097; doi:10.1007/s11121-025-01779-x)
Supplement: Supplementary file 4 — Supplementary file4 (PDF 133 KB) [file 11121_2025_1779_MOESM4_ESM.pdf]

Exploring Cultural Adaptations: A Scoping Review on Adolescent Mental Health and Substance Use Prevention Programs.
Prevention Science
Authors: Claudia Corpus-Espinosa, Isotta Mac Fadden*, María del Carmen Torrejón-Guirado, Marta Lima-Serrano
*** Corresponding author affiliation and email:** Social Sciences Faculty, Department of Sociology, Universidad de Salamanca, Francisco Tomás y Valiente Avenue, no., 37071, Salamanca, Spain, macfadden@usal.es

Supplemental File 4

Supplementary Table 3 Key adaptation steps and descriptions

Step name	Step description
**1. Local needs assessment.**	Identify risk and protective factors and target audience´s perceptions influencing health outcomes through gathering new data (e.g., community consultation and formative research) or existing data (e.g., literature reviews). Triangulating from multiple sources is considered best practice.<br>Gather data on resources for project development, and community organizations or groups working to enhance youth health.<br>The health landscape and adolescent needs are ever-changing, necessitating regular review and updates of this section to ensure ongoing improvement of the intervention.
**2. Program selection.**	Collect information on relevant existing evidence-based interventions.<br>Select EBIs aligned with the community's needs, resources, and context based on the local assessment.<br>Consider several factors such as age, ethnicity, language, duration, cost, staffing needs, outcomes, training availability, evidence of effectiveness, prior adaptation, etc. when selecting interventions.
**3. Understanding program's curriculum.**	Understand the program's theoretical foundation, including precursor ideas, target population, and the health issue addressed.<br>Identify and understand the core components, encompassing main intervention concepts, health education messages, outcomes, objectives, and desired impact.
**4. Advisory group establishment.**<br>**4.1 Experts.**<br>**4.2 Target population /Stakeholders.**	Incorporate feedback from diverse perspectives including youth, community figures (e.g., parents, teachers, leaders), and other stakeholders.<br>Review materials for language, verb tense, appropriateness, cultural relevance, and social validity to identify positive features and areas for improvement.<br>The advisory group helps guide and co-design program adaptation, while linking adolescents, project staff, local contacts, and stakeholders is crucial.

Authors: Claudia Corpus-Espinosa, Isotta Mac Fadden*, María del Carmen Torrejón-Guirado, Marta Lima-Serrano
*** Corresponding author affiliation and email:** Social Sciences Faculty, Department of Sociology, Universidad de Salamanca, Francisco Tomás y Valiente Avenue, no., 37071, Salamanca, Spain, macfadden@usal.es

**5. First draft of initial adaptation changes.**	Based on previous steps, discuss, and agree on whether to adapt the original program, and if so, what changes will be made.<br>Preliminary adaptations may include translation, subtitling, or dubbing, along with minor adjustments to examples, songs, vocabulary, idioms, and information for cultural alignment. Back translation is essential to maintain fidelity to the original content.<br>Collaborate with the advisory group, including original authors, to preserve the theory and core components of the intervention.<br>Diverse participation, including various genders, ages, educational backgrounds, etc., is crucial at this stage.<br>As a result, an initial draft of the program will be created, then evaluated to identify barriers and successes.
**6. Staff selection and training.**	Identify training participants: experts, stakeholders, or the target population (adolescents).<br>Select and train staff under certified professionals' or program authors' guidance to ensure safe, engaging experiences for participants and high-quality implementation.<br>Adapt training as needed.
**7. Pilot study.**	Implement the adapted intervention on a smaller scale for program refinement before full-scale implementation.<br>Identify target audience and stakeholders for intervention feedback.<br>Define information collection tools for stakeholders and target audience, e.g., focus groups, interviews, workshops, etc.<br>Decide if the audience will receive the entire adapted program or just excerpts.<br>Piloting should gather data on recruitment, retention rates, intervention fidelity, social validation (feasibility, acceptability, satisfaction, perceived usefulness), and barriers.<br>Allocate ample time and resources, to refine the intervention during the pilot phase.
**8. Enhanced cultural adaptation.**	Refine program elements requiring adaptation based on preceding step results through collaboration with the target audience, stakeholders, and original authors.<br>The result will be the adapted intervention ready for full implementation.
**9. Implementation.**	Develop an implementation plan with the target community and stakeholders based on prior step results.<br>Scale up the implementation of the adapted intervention.<br>Promote long-term sustainability.
**10. Evaluation and monitoring.**	Monitoring and evaluation gather ongoing information to assess if the adapted intervention achieves objectives and impacts health outcomes.<br>Plan program monitoring and evaluation.<br>Monitor should aim to assess the fidelity and quality.

Authors: Claudia Corpus-Espinosa, Isotta Mac Fadden*, María del Carmen Torrejón-Guirado, Marta Lima-Serrano
*** Corresponding author affiliation and email:** Social Sciences Faculty, Department of Sociology, Universidad de Salamanca, Francisco Tomás y Valiente Avenue, no., 37071, Salamanca, Spain, macfadden@usal.es

	There are multiple study types to evaluate program´s efficacy, but an RCT is recommended as the most robust design.<br>Evaluate the social validity of the adapted intervention.<br>Analyze factors for impact and sustainability.
**11. Dissemination.**	If the adapted intervention proves to be effective, implement a large-scale dissemination strategy.
